# Explainable Machine Learning for Prediction of Future High-Severity States Using Longitudinal APACHE-II Trajectories: Internal Validation and Cross-Cohort Portability Assessment

**DOI:** 10.3390/diagnostics16193118

**Published:** 2026-09-25

**Authors:** İrem Akpolat, Fatma Çelik

**Affiliations:** 1 Department of Nutrition and Dietetics, İstanbul Okan University, Istanbul 34959, Türkiye; 2 Department of Nutrition and Dietetics, Faculty of Health Sciences, Biruni University, Istanbul 34015, Türkiye; fcelik@biruni.edu.tr

**Keywords:** intensive care unit (ICU), total parenteral nutrition (TPN), longitudinal machine learning, APACHE-II, severity-state prediction, explainable artificial intelligence (XAI)

## Abstract

**Background**: Longitudinal severity assessment may provide more informative risk stratification than reliance on a single admission score in intensive care. This study developed and internally validated an explainable machine learning framework for predicting a subsequent APACHE-II-defined high-severity state using repeated APACHE-II assessments in intensive care unit (ICU) patients receiving total parenteral nutrition (TPN). The endpoint was defined as an APACHE-II score of ≥ 20 at the final assessment (T10), while predictor information was restricted to measurements obtained at T0–T9. **Methods**: A retrospective institutional cohort of 844 adult ICU patients was analyzed, of whom 214 (25.4%) met the predefined high-severity endpoint. Four principal feature representations were evaluated: admission APACHE-II, longitudinal APACHE-II information combining the raw T0–T9 sequence with derived trajectory descriptors, longitudinal laboratory information, and multimodal combinations including baseline characteristics. Six machine learning algorithms were compared using stratified five-fold cross-validation and independent hold-out testing. Additional analyses included simpler APACHE-II comparators, sequential observation truncation, nested cross-validation, calibration assessment, threshold sensitivity analysis, and SHAP-based model interpretation. Because equivalent longitudinal APACHE-II measurements and an equivalent endpoint were unavailable in eICU, a separate harmonized XGBoost model was used only for exploratory cross-cohort portability assessment. **Results**: The longitudinal APACHE-II CatBoost model achieved the highest performance, with a cross-validated ROC-AUC of 0.924 and an independent hold-out ROC-AUC of 0.921 (95% CI: 0.878–0.960). Hold-out calibration was good (Brier score = 0.095, calibration intercept = − 0.03, slope = 0.98), and nested cross-validation yielded a pooled out-of-fold ROC-AUC of 0.908 and a mean outer-fold ROC-AUC of 0.920 ± 0.038. The latest APACHE-II assessment alone (T9; ROC-AUC = 0.553), change from baseline (0.576), ordinal slope (0.539), and logistic regression using engineered APACHE-II descriptors (0.633) performed substantially below the full longitudinal CatBoost model. Sequential truncation showed that discrimination was retained after removing later observations, although performance varied non-monotonically across truncated sequences. SHAP analysis showed that multiple APACHE-II observations contributed prominently to model predictions, with trajectory-derived descriptors providing complementary contributions. Adding laboratory trajectories or baseline characteristics did not improve discrimination over the longitudinal APACHE-II representation. **Conclusions**: Nonlinear modeling of repeated APACHE-II assessments provided strong internal discrimination of a subsequent APACHE-II-defined high-severity state in this TPN-treated ICU cohort and substantially outperformed single-score and simpler longitudinal comparators. However, the predictors and endpoint share the APACHE-II construct, observation indices were sequential rather than standardized clock-time intervals, and the primary model could not undergo conventional external validation in eICU. These findings therefore support the internal predictive value of longitudinal APACHE-II modeling for this specific severity-state task and warrant prospective multicenter evaluation using standardized timing and independent clinical outcomes.

## 1. Introduction

### 1.1. Burden of Critical Illness in Intensive Care Units

Critical illness remains a leading cause of morbidity, mortality, and healthcare expenditure worldwide. Intensive care units (ICUs) provide continuous monitoring and advanced life support for patients with severe physiological instability and life-threatening conditions [1,2].

The clinical course of critically ill patients is highly dynamic and unpredictable; without timely intervention, rapid clinical deterioration may result in irreversible organ damage or death. Despite continuous monitoring and repeated clinical assessments, the early recognition of deterioration remains challenging because of the complex and heterogeneous nature of critically ill patients [3]. Among these patients, those requiring total parenteral nutrition (TPN) constitute a clinically more complex subgroup in whom oral or enteral nutrition is either not feasible or insufficient [4]. Consequently, there is growing interest in computational approaches capable of identifying high-risk patients before overt clinical deterioration occurs [5].

Early-warning systems are designed to identify physiological changes associated with impending clinical deterioration and thereby facilitate earlier clinical assessment and intervention [5,6]. Widely used severity and organ-dysfunction scoring systems, including the Acute Physiology and Chronic Health Evaluation II (APACHE-II), Sequential Organ Failure Assessment (SOFA), and Simplified Acute Physiology Score II (SAPS II), provide standardized approaches for quantifying illness severity and estimating prognosis in critically ill patients [7,8,9].

Beyond its clinical impact, critical illness places a substantial burden on healthcare systems through high demands for specialized personnel, advanced monitoring, mechanical ventilation, renal replacement therapy, and other resource-intensive interventions. Accurate identification of patients at risk may improve ICU triage, optimize resource allocation, and support more efficient healthcare planning [10,11].

The widespread adoption of electronic health records and digital monitoring systems has created unprecedented opportunities for data-driven critical care. Modern ICUs routinely generate large volumes of longitudinal clinical data, including physiological measurements, laboratory results, treatments, and severity assessments. These data provide a rich foundation for artificial intelligence and machine learning approaches capable of transforming routine clinical information into clinically actionable early-warning systems for proactive patient management [12,13].

### 1.2. APACHE-II as a Severity Assessment Tool

Accurate severity assessment is fundamental to intensive care medicine because it supports prognosis estimation, treatment prioritization, risk stratification, and resource allocation. Among available severity scoring systems, the Acute Physiology and Chronic Health Evaluation II (APACHE-II) remains a widely used tool for assessing illness severity in adult ICU patients [7]. APACHE-II combines acute physiological variables with age and chronic health information to generate an overall severity score [7]. Its established clinical use and prognostic performance have supported its application for severity and outcome assessment across heterogeneous ICU populations [7,14,15].

APACHE-II and related severity-scoring systems have also been extensively evaluated as tools for risk stratification and outcome prediction in critical-care research. Their interpretation and application extend beyond individual prognosis to benchmarking patient populations and evaluating performance across clinical settings [16,17].

Despite these strengths, conventional use of APACHE-II has important limitations when the objective is to characterize subsequent changes in patient status. The original APACHE-II system was developed as a severity-of-disease classification and prognostic framework rather than as a longitudinal model of evolving disease trajectories [7]. APACHE-II is therefore commonly interpreted from individual or admission-period assessments, even though physiological status may change substantially during an ICU stay. Comparisons between admission and subsequent APACHE-II assessments demonstrate that the timing of severity assessment can influence prognostic information [18], while broader evaluations of critical-care scoring systems emphasize the limitations of relying on static severity measurements to represent an evolving clinical course [19].

This distinction is particularly relevant in critically ill patients, whose physiological status may evolve in response to disease progression, treatment, and complications. Dynamic modeling of ICU data has demonstrated that longitudinal information can capture changes in patient risk that are not represented by isolated baseline observations [20]. More specifically, studies evaluating repeated APACHE-II measurements have shown that dynamic APACHE-II assessment can provide prognostic information beyond a single static evaluation [21]. These observations provide the rationale for investigating whether repeated APACHE-II assessments can be represented as longitudinal trajectories and analyzed using machine-learning methods to discriminate a subsequent APACHE-II-defined high-severity state.

### 1.3. Machine Learning in Intensive Care

The increasing adoption of electronic health records and large critical care databases has accelerated the application of machine learning in intensive care medicine. Compared with conventional statistical approaches, machine learning algorithms can model complex nonlinear relationships, high-dimensional feature interactions, and temporal patterns within clinical data, enabling more accurate prediction of patient deterioration and supporting data-driven clinical decision-making [22,23].

Early prediction models primarily relied on conventional statistical methods such as Logistic Regression and Cox proportional hazards models because of their simplicity and interpretability. Although these approaches remain valuable for clinical risk estimation, their assumptions of linearity and proportional hazards limit their ability to represent the complex and evolving nature of critical illness.

Recent advances have shifted attention toward tree-based ensemble learning methods, which are well suited to heterogeneous clinical data. Algorithms such as Random Forest, XGBoost, LightGBM, and CatBoost have demonstrated strong performance across a range of intensive care applications, including mortality prediction, sepsis detection, and organ dysfunction assessment [24,25,26,27,28,29,30]. In addition to their predictive accuracy, these models integrate effectively with explainable artificial intelligence techniques, particularly SHapley Additive exPlanations (SHAP), enabling transparent interpretation of model predictions and facilitating clinical trust [31].

Deep learning architectures, including Long Short-Term Memory (LSTM) networks, Gated Recurrent Units (GRUs), and Transformer-based models, have also shown promise for modeling sequential electronic health records and physiological time-series data [32,33,34,35,36,37]. However, these approaches generally require substantially larger datasets, greater computational resources, and often provide less transparent decision-making than tree-based methods, which may limit their routine clinical implementation.

Despite these advances, important gaps remain. Most studies have focused on mortality prediction, sepsis detection, or organ failure, whereas relatively few have investigated subsequent APACHE-II-defined high-severity states using repeated APACHE-II assessments collected throughout ICU admission [28,29,30,38,39]. This limitation is particularly evident when evaluating longitudinal patterns of disease severity in critically ill patients receiving total parenteral nutrition, whose clinical trajectories may be more complex. Furthermore, although internal validation is increasingly common, relatively few studies have systematically evaluated model portability across independent healthcare environments using publicly available datasets such as eICU or MIMIC-IV [40,41]. Addressing these gaps may improve confidence in the robustness and clinical applicability of machine learning models for intensive care.

### 1.4. Explainable Artificial Intelligence in Healthcare

The increasing adoption of machine learning in healthcare has intensified the need for transparent and interpretable prediction models. Although advanced machine learning algorithms often achieve high predictive accuracy, many function as “black-box” systems, making it difficult for clinicians to understand how individual predictions are generated [42]. This lack of transparency can limit clinical acceptance, particularly in high-stakes settings where decisions directly influence patient safety and treatment outcomes.

Clinical trust is essential for the successful integration of artificial intelligence (AI) into healthcare. Physicians are less likely to rely on automated predictions that cannot be adequately explained, as clinical decisions require justification, accountability, and effective communication among multidisciplinary care teams [43]. In parallel, regulatory and ethical frameworks increasingly emphasize trustworthy AI, reinforcing the importance of explainable artificial intelligence (XAI) for model validation, transparency, and responsible clinical deployment.

Interpretability is especially important in intensive care, where rapidly changing patient conditions demand timely and well-informed decision-making. Understanding whether predictions are primarily driven by disease severity, physiological deterioration, laboratory findings, or chronic health conditions enables clinicians to better assess model reliability and incorporate AI-generated recommendations into routine practice [44]. Interpretable models may also reveal clinically meaningful patterns that contribute to improved understanding of disease progression.

Among current XAI techniques, SHapley Additive exPlanations (SHAP) has become one of the most widely adopted methods for interpreting machine learning models. Based on cooperative game theory, SHAP quantifies the contribution of individual features to model predictions while providing both patient-specific and global explanations of model behavior [31]. This dual-level interpretability makes SHAP particularly well suited for clinical decision-support applications.

Recent studies have successfully applied explainable machine-learning approaches to critical-care tasks including mortality prediction and ICU admission risk assessment [45,46]. Building on these advances, integrating SHAP with longitudinal APACHE-II representations enables transparent interpretation of how repeated severity assessments contribute to future risk estimation, thereby supporting clinically interpretable longitudinal risk-assessment frameworks for intensive care.

### 1.5. Limitations of Existing Literature

Despite substantial advances in machine learning for critical care, several important limitations remain. First, many prediction models are developed using single-center datasets, making them susceptible to institution-specific characteristics that may not generalize to other clinical settings [47]. Although internal cross-validation and independent hold-out testing provide valuable estimates of model performance, relatively few studies incorporate rigorous external evaluation across independent healthcare environments. Recent reviews have identified the lack of external validation as one of the most common barriers to the clinical translation of artificial intelligence models [48,49].

Second, existing research has focused predominantly on mortality prediction [38,39,50,51]. Although mortality is an important clinical endpoint, it is influenced by numerous factors beyond physiological deterioration, including treatment strategies, healthcare policies, and resource availability. Consequently, mortality prediction may provide limited support for timely clinical intervention. In contrast, forecasting future severity progression offers greater potential for early risk identification, proactive monitoring, and optimized resource allocation before patients reach a critical state.

Another important limitation concerns the use of APACHE-II. Despite being one of the most widely adopted severity scoring systems, it is typically incorporated into machine learning models as a single baseline variable, adjustment factor, or comparator rather than as a longitudinal representation of disease evolution [15,18,28]. Repeated APACHE-II assessments capture temporal changes in patient status that may better reflect treatment response and physiological deterioration; however, their predictive value for forecasting critical illness progression remains insufficiently explored [21]. Furthermore, few studies have systematically compared longitudinal severity representations with laboratory trajectories and multimodal longitudinal feature sets within a unified prediction framework.

These limitations highlight the need for clinically interpretable machine learning frameworks that leverage repeated severity assessments while incorporating rigorous validation and systematic evaluation of cross-cohort portability to improve the reliability and clinical applicability of early-warning systems in intensive care.

### 1.6. Study Objectives and Contributions

The primary objective of this study was to develop and evaluate an explainable machine learning framework for predicting a future high-severity state using longitudinal APACHE-II assessments collected during ICU monitoring. The primary endpoint was defined as a final APACHE-II score ≥ 20, while all preceding APACHE-II assessments (T0–T9) were restricted to the predictor space. Accordingly, the study evaluates whether patterns contained in earlier APACHE-II trajectories can discriminate a subsequent APACHE-II-defined high-severity state rather than predicting an independent clinical outcome such as mortality.

The central hypothesis was that repeated APACHE-II assessments contain predictive information beyond that provided by a single admission or recent severity score and that nonlinear modeling of the complete longitudinal trajectory can capture clinically relevant patterns that are not adequately represented by simple measures of change or linear trend. To test this hypothesis, raw APACHE-II sequences and trajectory-derived descriptors were evaluated using multiple machine learning algorithms and compared with admission-based and alternative clinical feature representations.

An important consideration of this design is the construct overlap between the predictors and outcome because both are derived from the APACHE-II scoring system. To evaluate whether model discrimination was driven predominantly by the most recent APACHE-II observation, additional analyses compared the full longitudinal framework with the latest available APACHE-II value alone, overall change, ordinal trajectory slope, and logistic regression models using the same longitudinal information. Sequential observation-truncation analyses were additionally performed to determine whether predictive performance was maintained when later APACHE-II assessments were excluded.

Explainable artificial intelligence using SHapley Additive exPlanations (SHAP) was incorporated to characterize the contribution of individual observations and trajectory descriptors to model predictions. These analyses were interpreted as explanations of model behavior rather than evidence of causal or independent clinical importance.

A secondary objective was to examine cross-cohort portability using the multicenter eICU Collaborative Research Database. Because eICU did not contain a directly equivalent longitudinal endpoint corresponding to the institutional definition of final APACHE-II ≥ 20, this analysis was designed as an exploratory assessment of feature compatibility, predicted-risk distribution shift, and transportability limitations rather than conventional external validation of the primary prediction task.

Collectively, the study evaluates whether longitudinal APACHE-II information provides incremental predictive value beyond individual severity assessments and simple trajectory summaries, while explicitly examining model stability, interpretability, and portability across heterogeneous critical-care data environments.

We hypothesized that nonlinear modeling of longitudinal APACHE-II trajectories would provide significantly greater discrimination of a subsequent APACHE-II-defined high-severity state than admission APACHE-II, the latest available APACHE-II assessment, simple trajectory descriptors such as change and slope, and conventional linear modeling of the same longitudinal information.

## 2. Materials and Methods

### 2.1. Study Design

**Figure 1:**
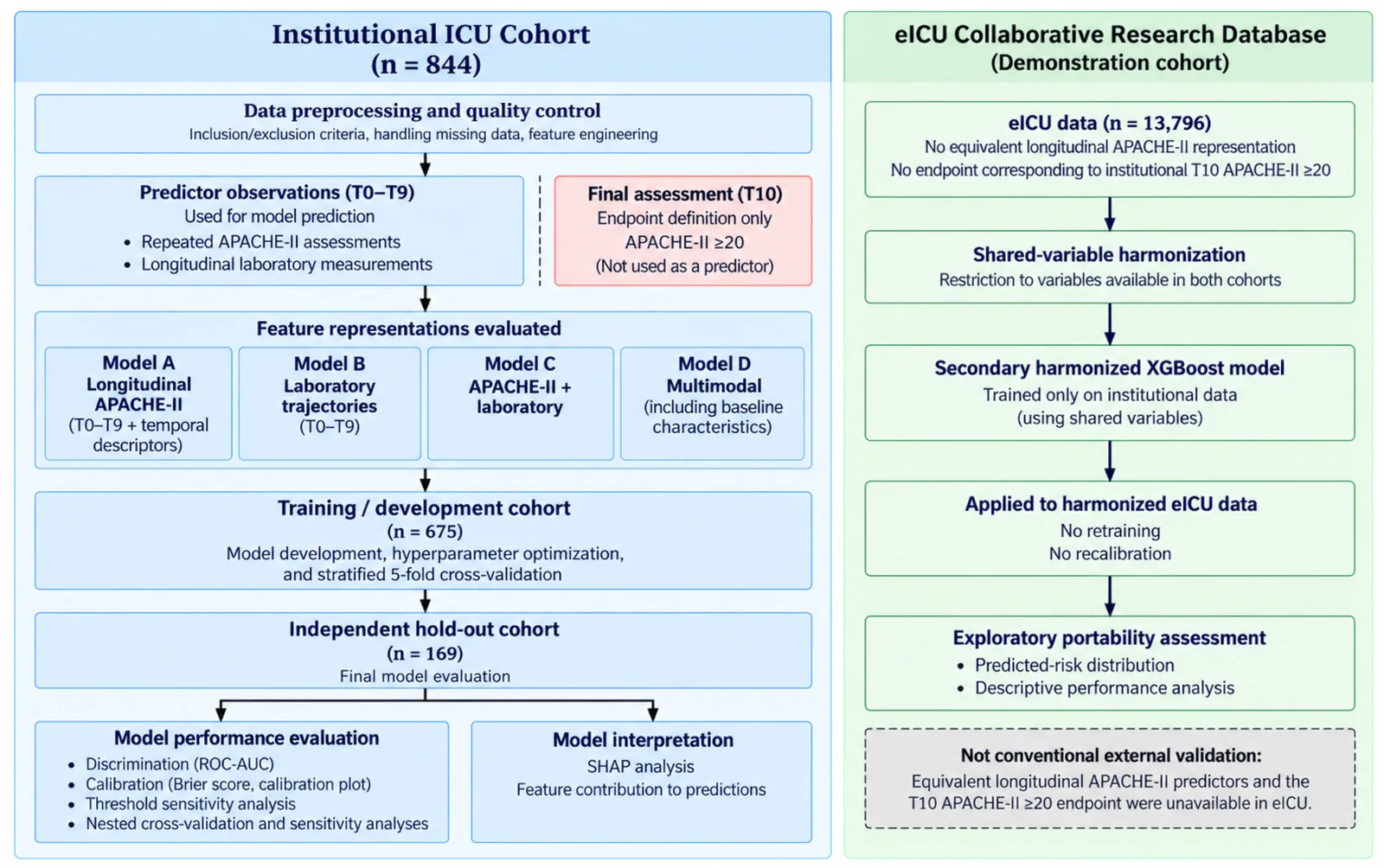
Overall study design and validation framework. The institutional ICU cohort was used for development and internal evaluation of models predicting a subsequent APACHE-II-defined high-severity state. APACHE-II assessments at T0–T9 and longitudinal laboratory measurements were used as predictors, whereas the final APACHE-II assessment at T10 was reserved exclusively for endpoint definition (APACHE-II ≥ 20). Model development, hyperparameter optimization, and selection were performed within the institutional training cohort, followed by evaluation on an independent hold-out cohort. Because equivalent longitudinal APACHE-II measurements and the institutional endpoint were unavailable in eICU, a separate harmonized XGBoost model based on shared variables was used only for exploratory cross-cohort portability assessment.

This retrospective multicohort study developed and evaluated an explainable machine learning framework for predicting a subsequent APACHE-II-defined high-severity state using repeated APACHE-II assessments and longitudinal laboratory measurements collected during ICU admission. The primary endpoint was defined as an APACHE-II score of ≥20 at the final assessment (T10), while only measurements obtained at T0–T9 were used as predictors. The overall study design is illustrated in Figure 1.

Two independent ICU cohorts were included. The institutional cohort was used for model development, hyperparameter optimization, internal validation, and independent hold-out testing. The publicly available eICU Collaborative Research Database Demonstration cohort was used only for an exploratory cross-cohort portability assessment. Because eICU does not contain an equivalent longitudinal APACHE-II representation or an endpoint corresponding to the institutional T10 APACHE-II ≥ 20 definition, the primary longitudinal Model A could not undergo conventional external validation.

Within the institutional cohort, longitudinal APACHE-II and laboratory measurements were preprocessed and transformed into structured feature representations before evaluation using multiple machine-learning algorithms. All computational analyses were performed in Python (Python Software Foundation, Wilmington, DE, USA). Data preprocessing, feature construction, model development, cross-validation, hyperparameter optimization, and performance evaluation were implemented using the relevant Python scientific-computing and machine-learning libraries, including NumPy (NumPy Developers), pandas (The pandas development team), and scikit-learn (scikit-learn developers). Gradient-boosting implementations included XGBoost (XGBoost Developers), LightGBM (Microsoft Corporation, Redmond, WA, USA), and CatBoost (Yandex LLC, Moscow, Russia). 

Model development and selection were restricted to the institutional training partition, followed by independent hold-out evaluation. A separate secondary XGBoost portability model, restricted to variables that could be harmonized between the institutional and eICU cohorts, was trained exclusively on institutional data and subsequently applied to eICU without retraining or recalibration. Model interpretation was performed using SHAP (SHapley Additive exPlanations; SHAP Developers) to quantify feature contributions to predictions from the selected institutional model. No commercial chemicals, reagents, cell lines, or study-specific laboratory instruments were used as part of the computational analyses reported in this study.

### 2.2. Data Sources

Two independent data sources were used in this study. The primary dataset consisted of an institutional intensive care unit (ICU) cohort used for model development and internal validation, while the publicly available eICU Collaborative Research Database Demonstration (eICU Demo) dataset was used for an exploratory assessment of cross-cohort portability. The inclusion of two independent cohorts enabled evaluation of model performance within the institutional population and assessment of framework transferability across heterogeneous clinical environments.

#### 2.2.1. Development Cohort

The development cohort comprised 844 adult ICU patients admitted to a tertiary-care hospital. Clinical data were collected retrospectively during routine patient care and anonymized before analysis. Available demographic and baseline clinical variables included age, sex, body weight, height, body mass index (BMI), diabetes mellitus, chronic kidney disease, hypertension, coronary artery disease, chronic obstructive pulmonary disease, and hepatitis history.

For each patient, repeated APACHE-II assessments and serial laboratory measurements collected throughout ICU admission were available. Laboratory biomarkers included alanine aminotransferase (ALT), albumin, alkaline phosphatase (ALP), aspartate aminotransferase (AST), total bilirubin, C-reactive protein (CRP), gamma-glutamyl transferase (GGT), glucose, calcium, creatinine, magnesium, and potassium. These longitudinal measurements were subsequently transformed into predictive feature representations as described in Section 2.5.

Repeated APACHE-II assessments were indexed sequentially as T0–T9, with T0 representing the initial assessment and T10 reserved exclusively for outcome definition. Because assessment timing depended on routine clinical practice and patient condition, these observation points represent sequential clinical evaluations rather than fixed time intervals. To preserve temporal separation between predictors and outcome, only measurements obtained before the final assessment were included in model development.

Eligible patients were 18 years of age or older, admitted to the ICU, and had complete longitudinal APACHE-II assessments, repeated laboratory measurements, and sufficient follow-up to determine the final APACHE-II outcome.

The institutional source dataset contained 844 eligible patient records. All 844 patients had complete APACHE-II assessments from T0 through T10, including the endpoint-defining T10 assessment; therefore, no patients were excluded because of incomplete APACHE-II sequences or missing endpoint information. No duplicate patient identifiers were identified, and no patients were removed because of incomplete laboratory trajectories. Consequently, all 844 records were retained in the final analytical cohort. The institutional cohort-selection and data-partitioning process is summarized in Supplementary Figure S1.

#### 2.2.2. Cross-Cohort Portability Assessment Dataset

An exploratory cross-cohort portability assessment was conducted using the eICU Collaborative Research Database Demonstration (eICU Demo, v2.0), a publicly available subset of the eICU Collaborative Research Database containing de-identified ICU records collected from multiple hospitals across the United States [52]. The dataset is intended primarily for methodological development, algorithm evaluation, and reproducible research.

Adult ICU patients with available APACHE-related severity information, repeated laboratory measurements, and outcome data were identified for the portability analysis. Clinical variables were harmonized with those of the institutional cohort by aligning common demographic characteristics, laboratory measurements, measurement units, and feature representations. Only variables available in both cohorts were retained to ensure methodological consistency.

The eICU Demo cohort was used exclusively for cross-cohort portability assessment and was not involved in preprocessing parameter estimation, feature engineering, feature selection, model development, hyperparameter optimization, or model selection. A secondary XGBoost portability model based on harmonized variables was trained solely on the institutional cohort and subsequently applied to the eICU Demo cohort without retraining or recalibration. The characteristics of both cohorts are summarized in Table 1.

The eICU Demo source contained 13,796 ICU records. After application of the study-specific adult eligibility and variable-availability criteria, 2520 records constituted the candidate portability cohort, of which 2203 had the shared variables required for the final harmonized analysis.

As shown in Table 1, the institutional and eICU cohorts differed substantially in demographic characteristics, data availability, and outcome definitions, highlighting the challenges associated with evaluating model portability across heterogeneous clinical environments.

### 2.3. Outcome Definition

#### 2.3.1. Primary Endpoint: Future APACHE-II-Defined High-Severity State

The primary endpoint was a future high-severity state defined according to the final available APACHE-II assessment (T10) during the analyzed ICU monitoring sequence. Patients with a final APACHE-II score ≥ 20 were classified as having reached the predefined high-severity state, whereas those with a final APACHE-II score < 20 were classified as not having reached this state. This threshold was selected to distinguish patients with substantially greater physiological severity and expected clinical risk based on the established interpretation of APACHE-II scores [7,15,18].

The binary outcome was therefore defined as:

Y_i_ = 1 if APACHE-II_i_,T10 ≥ 20; otherwise Y_i_ = 0.

Among the 844 patients in the institutional cohort, 214 (25.4%) met the predefined endpoint and 630 (74.6%) did not.

Importantly, this endpoint should not be interpreted as a direct measure of clinical deterioration from baseline. Because classification was based on the absolute final APACHE-II value, a patient could meet the high-severity endpoint despite having a stable or decreasing APACHE-II trajectory. The prediction task therefore evaluates discrimination of a subsequent high-severity state, rather than requiring evidence of worsening relative to an earlier APACHE-II assessment.

The final APACHE-II assessment (T10) and all information derived from it were excluded from predictor construction. Only APACHE-II observations T0–T9 and other eligible information available before the endpoint assessment were used for model development.

#### 2.3.2. Sequential Prediction Framework and Temporal Information Availability

For each patient, the first ten APACHE-II observations in the analyzed longitudinal sequence (T0–T9) were used as predictor measurements, whereas the subsequent assessment (T10) was reserved exclusively for endpoint definition. Thus, the notation T0–T10 represents the ordered sequence of available APACHE-II assessments and should not be interpreted as fixed or equally spaced clock-time intervals.

Actual timestamps corresponding to individual APACHE-II observations were not available in a form that permitted reliable reconstruction of patient-specific elapsed intervals across the complete cohort. Consequently, a uniform prediction horizon in hours or days could not be established retrospectively. For this reason, the present study describes the task as prediction of a subsequent APACHE-II-defined high-severity state rather than assigning a fixed early-warning interval.

To examine whether predictive performance depended disproportionately on observations closest to the endpoint, sequential observation-truncation sensitivity analyses were performed. Models were reconstructed using progressively shortened predictor sequences from T0–T9 through T0–T3 while maintaining T10 exclusively as the endpoint-defining assessment. These analyses quantify the robustness of prediction to removal of later observations but should not be interpreted as fixed 24-, 48-, or 72-h prediction horizons.

#### 2.3.3. Outcome Separation and Leakage Prevention

To prevent information leakage, APACHE-II observations T0–T9 were used exclusively for predictor construction, whereas T10 was reserved solely for defining the high-severity endpoint. T10 and all features derived from it were excluded from model development. Although this ensured separation between predictor and outcome information, both were derived from the APACHE-II scoring system, resulting in inherent construct overlap. This issue was further examined in sensitivity analyses comparing the longitudinal model with simpler APACHE-II predictors and progressively truncated observation sequences.

### 2.4. Data Preprocessing

**Figure 2:**
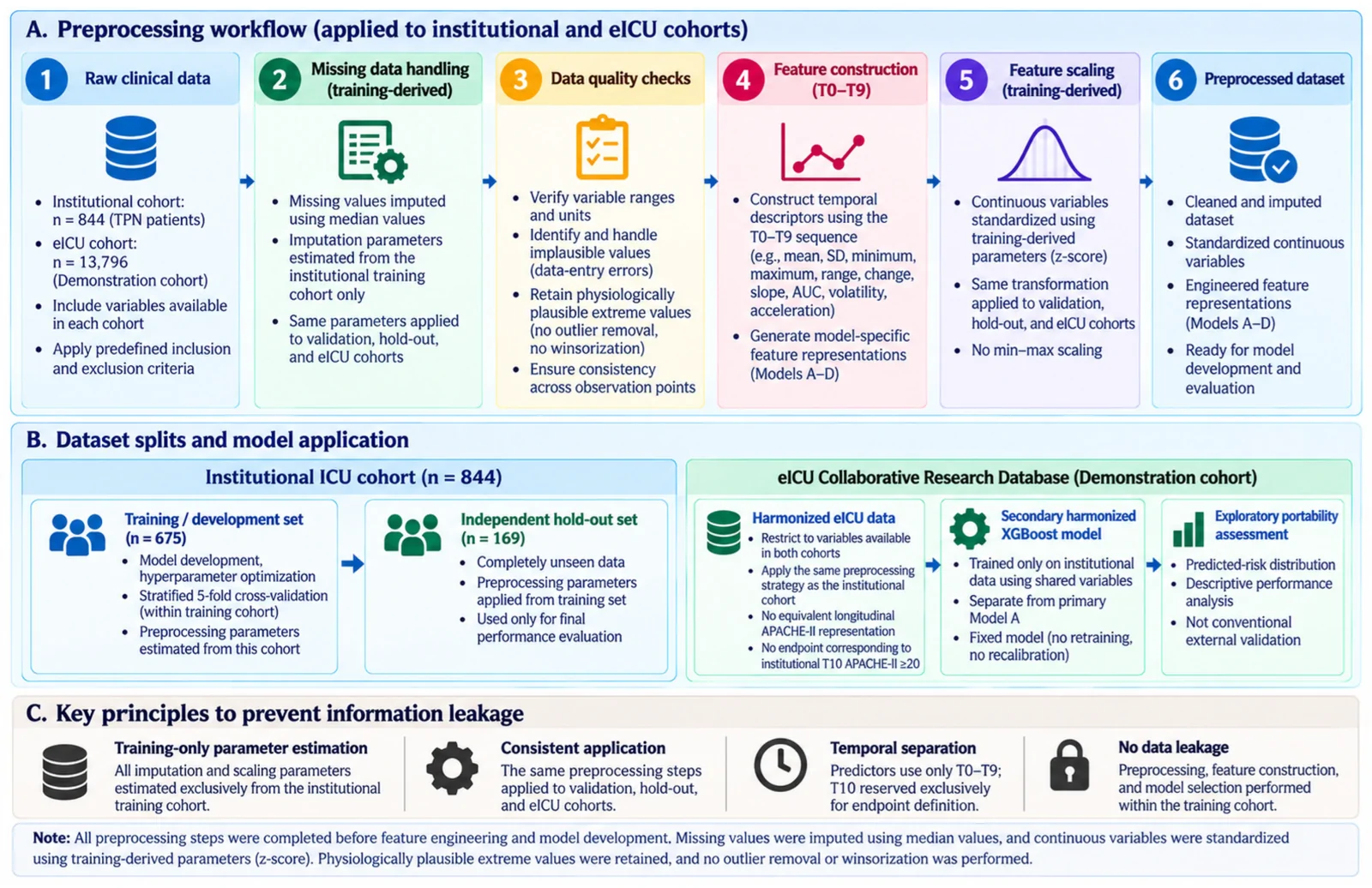
Standardized data preprocessing and leakage-prevention workflow. Preprocessing parameters were estimated from training data and subsequently applied to held-out observations. The final APACHE-II assessment (T10) was reserved exclusively for endpoint definition and excluded from predictor construction.

Before model development, clinical variables underwent a standardized preprocessing procedure designed to ensure data consistency and minimize information leakage (Figure 2). Missing continuous values were handled using median imputation, with imputation parameters estimated from the institutional training data and subsequently applied to held-out data. Missingness indicators were additionally incorporated during preprocessing to retain information regarding the presence of missing observations. Continuous features were standardized using parameters estimated from the training data. These preprocessing operations were implemented within the model pipeline so that parameter estimation occurred exclusively from the relevant training data during model development and cross-validation.

Extreme clinical measurements were retained when considered physiologically plausible because such values may represent genuine disease severity in critically ill patients rather than erroneous observations. Data-quality checks were performed to identify implausible values and ensure consistency before feature construction. In the final analytical cohort, APACHE-II observations T0–T9 were complete, while missingness among processed laboratory features was low.

For the exploratory cross-cohort portability assessment, variables shared between the institutional and eICU cohorts were harmonized with respect to variable definitions, laboratory nomenclature, measurement units, and categorical encoding. Only compatible variables were retained for harmonized analyses. The eICU cohort was not used for primary model training, hyperparameter optimization, or selection.

The institutional cohort contained 214 patients (25.4%) meeting the predefined high-severity endpoint and 630 (74.6%) not meeting the endpoint. Class weighting was applied where supported by the evaluated algorithms to account for this imbalance, with weights determined from the training data.

Information leakage was controlled throughout the analytical workflow. The final APACHE-II assessment (T10), which defined the primary endpoint, and all information derived from it were excluded from predictor construction. Preprocessing parameters were learned exclusively from training data, while hyperparameter optimization and model selection were conducted within the training partition using cross-validation. The independent institutional hold-out set remained isolated from model fitting and threshold optimization.

### 2.5. Clinical Feature Representation

The proposed framework represented patient status using repeated APACHE-II assessments together with longitudinal laboratory measurements collected during ICU admission. APACHE-II observations T0–T9 formed the predictor sequence, while T10 was reserved exclusively for definition of the subsequent APACHE-II-based high-severity endpoint. As established in Section 2.3, T0–T10 denote sequential clinical observations rather than fixed or equally spaced clock-time intervals.

**Figure 3:**
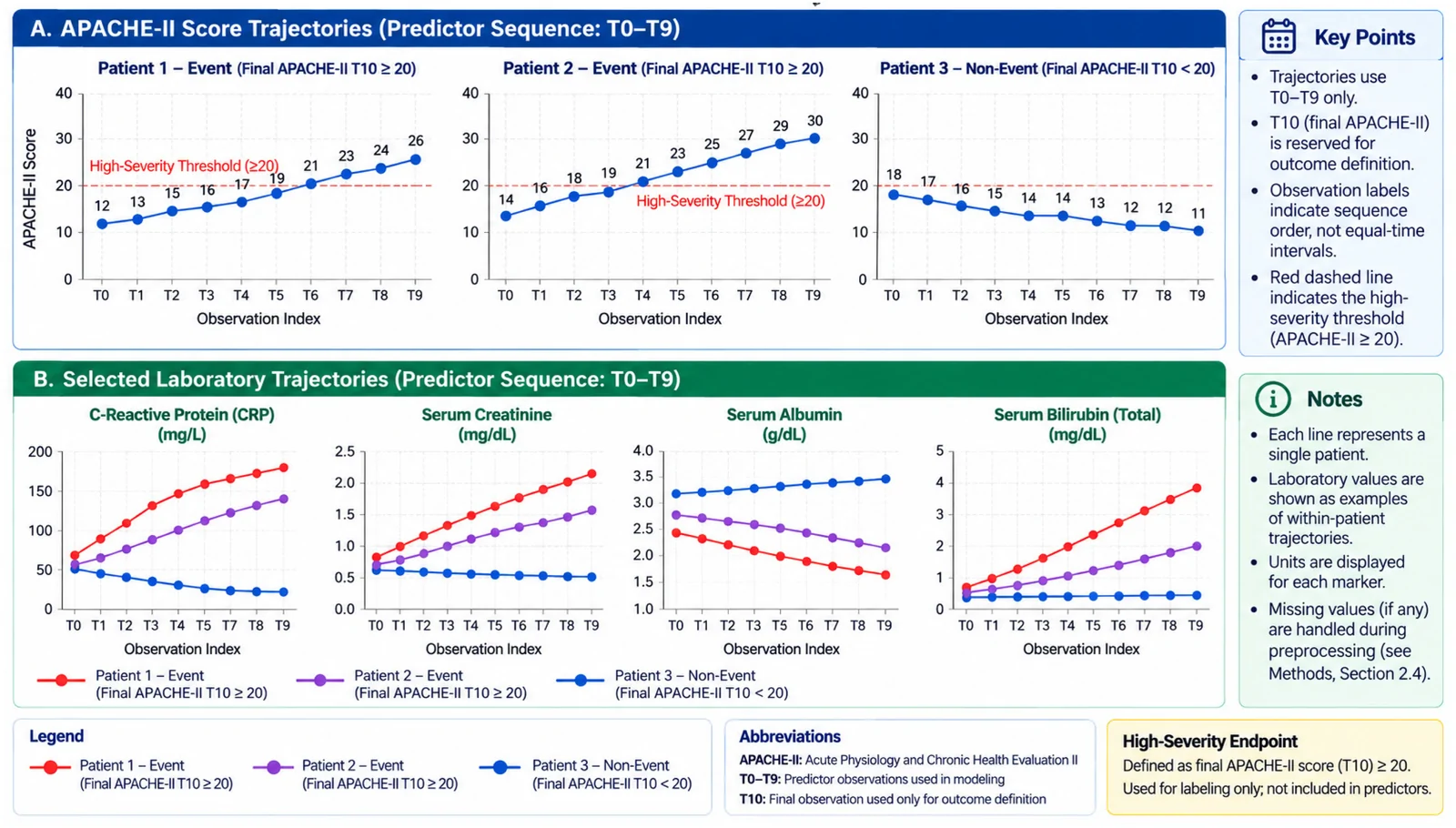
Representative longitudinal clinical trajectories before endpoint assessment. Examples of sequential APACHE-II assessments and selected laboratory measurements illustrate within-patient variation across the predictor observation sequence. Observation labels represent measurement order rather than standardized elapsed-time intervals.

Twelve laboratory biomarkers were evaluated: albumin, ALT, AST, ALP, total bilirubin, CRP, GGT, glucose, calcium, creatinine, magnesium, and potassium. These measurements provided complementary information regarding changes in physiological status during ICU monitoring. Representative APACHE-II and laboratory trajectories are shown in Figure 3.

The longitudinal APACHE-II sequence, laboratory information, and baseline patient characteristics were subsequently organized into alternative feature representations to determine the relative predictive contribution of each information source.

### 2.6. Feature Engineering

**Figure 4:**
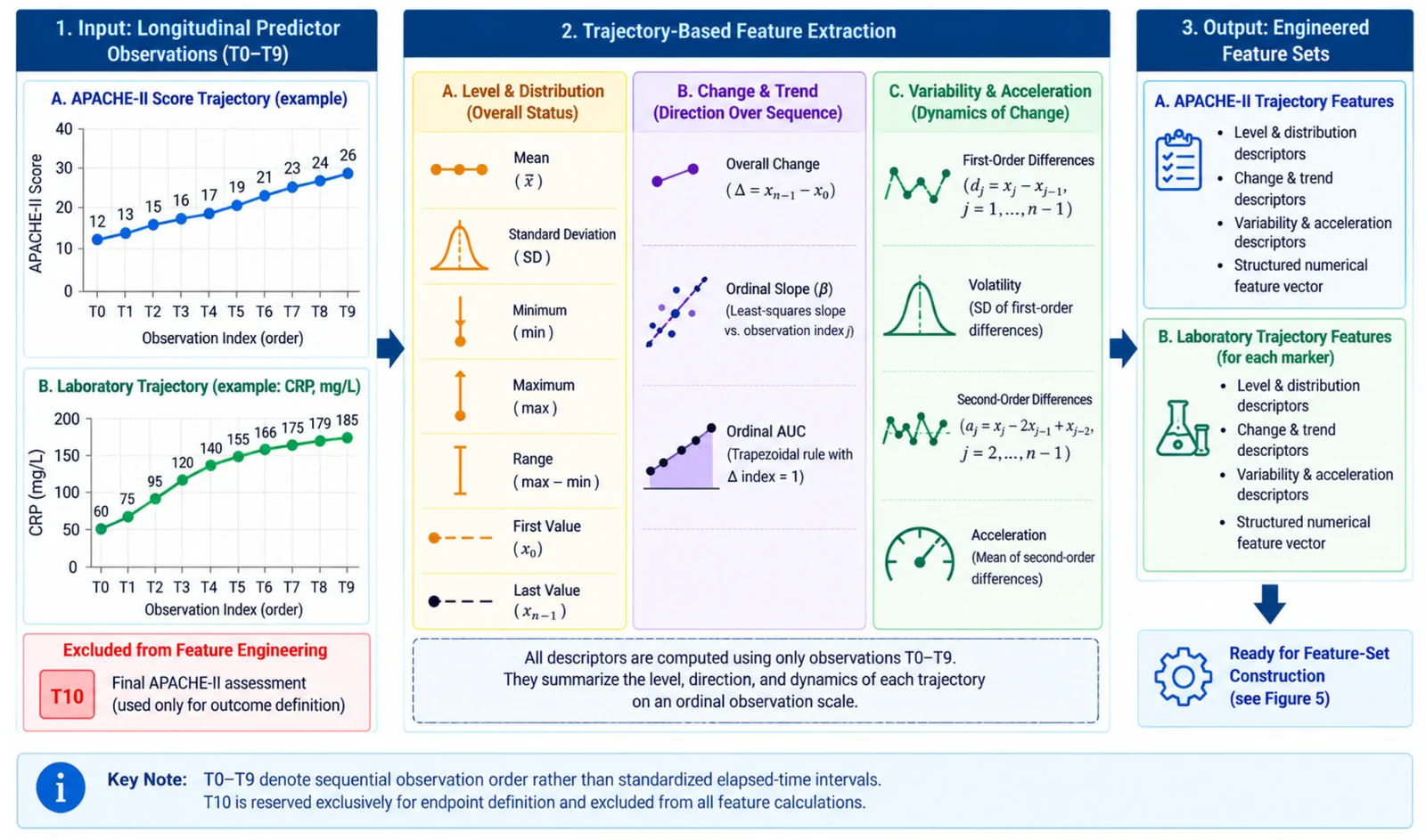
Longitudinal feature-engineering framework. Sequential APACHE-II and laboratory observations were transformed into statistical and ordinal trajectory descriptors, including overall level, variability, change, slope, observation-index AUC, volatility, and acceleration. The endpoint-defining APACHE-II assessment T10 was excluded from all feature construction.

Longitudinal observations were transformed into structured numerical descriptors suitable for machine learning, as illustrated in Figure 4. Feature extraction was performed independently for the APACHE-II sequence and for each laboratory trajectory using only predictor observations available before T10.

For each longitudinal sequence *x*_0_,…,*x_n_*_−1_ standard descriptors included the mean, standard deviation, minimum, maximum, range, first observation, last observation, and overall change:

Δ*x* = *x_n_*_−1_ − *x*_0_

The ordinal trajectory slope was estimated by least-squares regression against the sequential observation index *j*:

$$\beta =\frac{\sum_{j=0}^{n-1}(\left.j-\bar{j}\right)\left(x_{j}-\bar{x}\right)}{\sum_{j=0}^{n-1}(\left.j-\bar{j}{\left.)\right.}^{2}\right.}$$

and the observation-index area under the trajectory was calculated using trapezoidal integration:

$$\mathrm{AU}C_{\mathrm{ord}}=\sum_{j=0}^{n-2}\frac{x_{j}+x_{j+1}}{2}.$$

Accordingly, these quantities summarize trajectory behavior across ordered observations and should not be interpreted as clock-time rates or time-integrated physiological exposure.

Successive changes were defined as

*d_j_* = *x_j_* − *x_j_*_−1_, *j* = 1,…,*n* − 1,

and volatility was calculated as the standard deviation of these first-order differences. Second-order changes were defined as

*a_j_* = *d_j_* − *d_j_*_−1_ = *x_j_* − 2*x_j_*_−1_ + *x_j_*_−2_,

with acceleration represented by their mean:

$$A=\frac{1}{n-2}\sum_{j=2}^{n-1}a_{j}.$$

An independent implementation audit performed during revision reproduced all stored APACHE-II-derived trajectory features within numerical tolerance, confirming consistency between the implemented calculations and the corrected mathematical definitions.

### 2.7. Feature Sets and Incremental Comparator Analysis

Four principal feature representations were evaluated, as summarized in Table 2. The clinical baseline consisted of admission APACHE-II (T0). Model A combined the raw APACHE-II sequence T0–T9 with APACHE-II-derived trajectory descriptors. Model B contained longitudinal laboratory information and laboratory-derived trajectory descriptors. Model C combined Models A and B, whereas Model D additionally incorporated baseline characteristics, including age, sex, BMI, and available chronic conditions. T10 and all information derived from T10 were excluded from every predictor representation.

Because the primary endpoint was defined using APACHE-II, additional comparator analyses were performed to determine whether Model A provided information beyond a single recent severity assessment or simple trajectory summaries. The longitudinal CatBoost model was therefore compared with T9 alone, change from baseline (T9–T0), ordinal slope alone, T9 combined with change and slope, logistic regression using the raw T0–T9 sequence, logistic regression using engineered APACHE-II descriptors, and logistic regression combining the raw sequence with the engineered descriptors. These analyses were conducted as sensitivity analyses and did not modify the original primary model.

**Figure 5:**
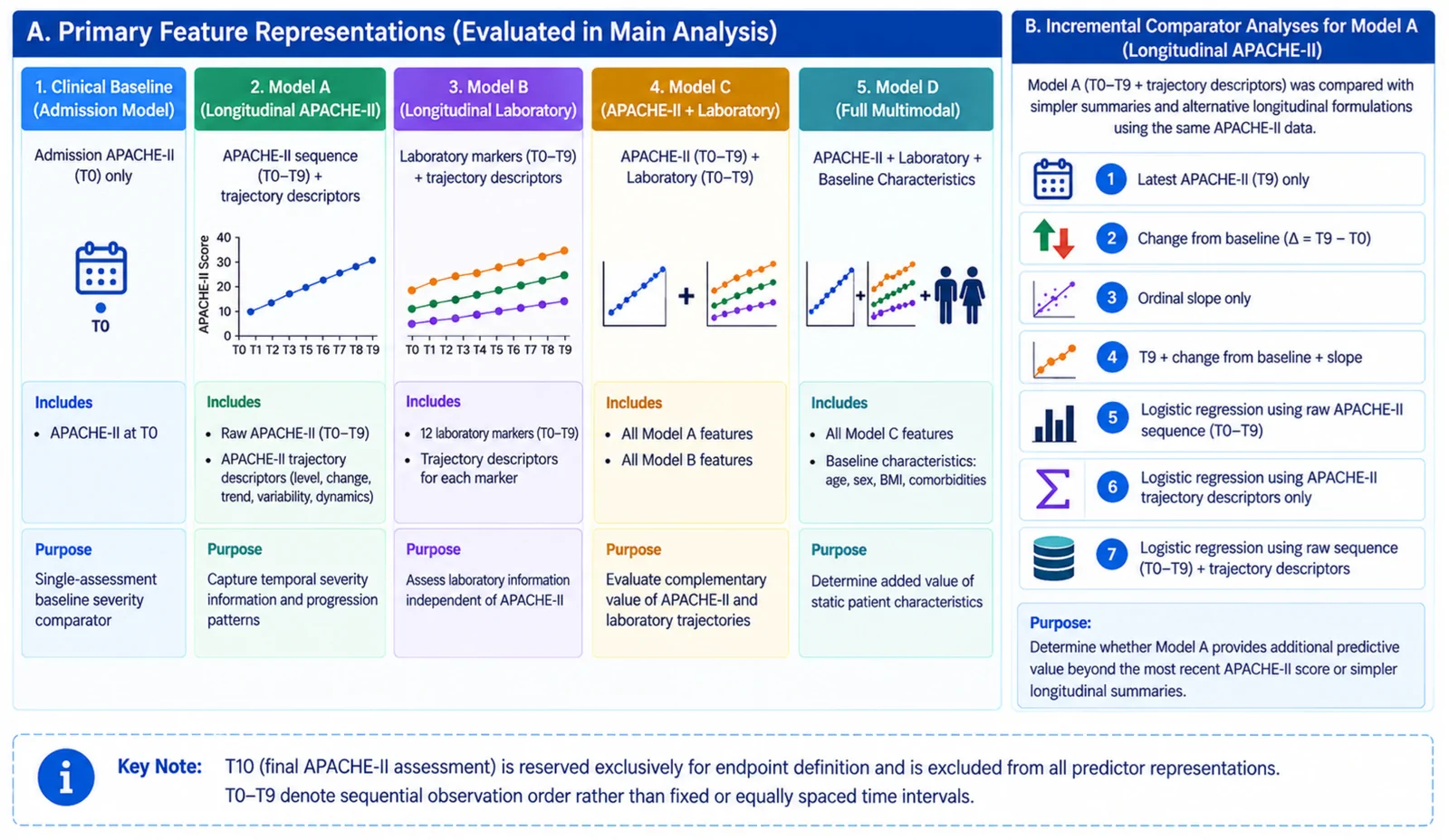
Feature representations and incremental-comparison framework. The primary analysis compared admission APACHE-II, longitudinal APACHE-II (Model A), longitudinal laboratory information (Model B), combined APACHE-II and laboratory information (Model C), and the full multimodal representation (Model D). Additional sensitivity analyses compared Model A with the latest APACHE-II observation and simpler longitudinal summaries to assess its incremental predictive value.

The relationships among the primary feature representations and the additional comparator analyses are summarized in Figure 5.

### 2.8. Machine Learning Development

Six classification algorithms were evaluated: Logistic Regression [53], Random Forest [24], Extra Trees [54], XGBoost [25], LightGBM [26], and CatBoost [27]. All algorithms were evaluated using the same institutional partition and corresponding feature representations.

The institutional cohort was divided once by stratified random sampling into a training/development set of 675 patients (80%) and an independent hold-out set of 169 patients (20%). Hyperparameter optimization and model selection were restricted to the training partition and performed using randomized search with stratified five-fold cross-validation, with ROC-AUC as the primary optimization criterion. Complete results for all six algorithms are provided in Supplementary Table S1, addressing potential bias from reporting only the best-performing algorithm.

For each primary feature representation, the model with the highest out-of-fold training ROC-AUC was selected for independent hold-out evaluation. CatBoost was selected for Model A. The final CatBoost configuration used 300 boosting iterations, a learning rate of 0.03, tree depth of 8, L2 regularization of 7, random strength of 0, and a border count of 64. The final operating threshold was selected exclusively from out-of-fold training predictions using the maximum Youden index and was fixed before evaluation of the hold-out cohort.

The original model-selection procedure used five-fold cross-validation but was not a fully nested cross-validation design. Therefore, an additional nested-CV robustness analysis was performed during revision for Model A. Five outer stratified folds were used for performance estimation, with three-fold inner cross-validation and 20 randomized-search iterations used independently within each outer-training fold for hyperparameter optimization. This supplementary analysis assessed potential model-selection optimism without modifying the original CatBoost model or its hold-out predictions. Fold-specific nested cross-validation results are provided in Supplementary Table S3.

### 2.9. Validation, Sensitivity, and Statistical Analysis

**Figure 6:**
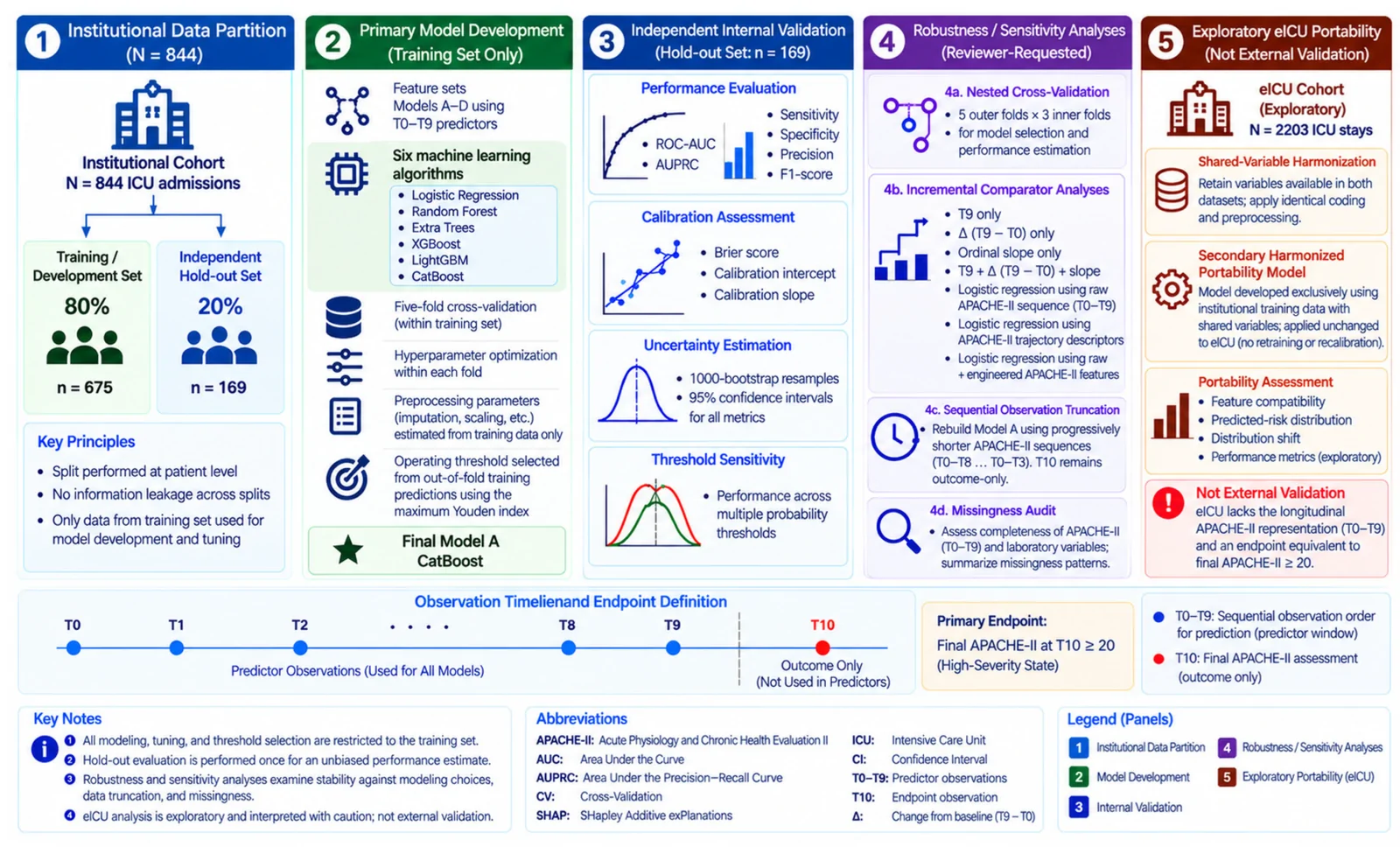
Model validation and robustness framework. Internal model development and independent hold-out evaluation, with additional nested cross-validation, sequential observation-truncation analysis, and exploratory cross-cohort portability assessment.

The evaluation framework is summarized in Figure 6. Internal validation comprised five-fold cross-validation within the training partition followed by independent hold-out testing. Discrimination was assessed using ROC-AUC, AUPRC, accuracy, sensitivity, specificity, precision, and F1-score. Calibration was evaluated using the Brier score, calibration intercept and slope, and reliability analysis. Bootstrap resampling (1000 iterations) provided 95% confidence intervals, and threshold sensitivity was evaluated around the training-derived operating threshold.

Robustness was further examined using nested cross-validation and sequential observation-truncation analysis. For the latter, Model A was reconstructed using progressively shortened APACHE-II sequences from T0–T9 to T0–T3, with T10 retained exclusively for endpoint definition. Because reliable timestamps were unavailable, this analysis assessed sensitivity to removal of later sequential observations, rather than fixed clock-time prediction horizons.

A missingness audit confirmed complete APACHE-II T0–T9 observations in all 844 patients and low laboratory-feature missingness (maximum 1.54%; Supplementary Table S2). Missing values were handled using training-derived median imputation as described in Section 2.4. SHAP was used to quantify model-level feature contributions and was not interpreted as evidence of causality.

Continuous baseline variables were compared using Welch’s independent-samples *t*-test and categorical variables using the χ^2^ test. All tests were two-sided, with *p* < 0.05 considered statistically significant.

Because eICU lacked an equivalent longitudinal APACHE-II representation and primary endpoint, it was used only for exploratory cross-cohort portability assessment, not conventional external validation.

### 2.10. Ethical Considerations

The study was conducted in accordance with the Declaration of Helsinki and was approved by the Ethics Committee of Dicle University Faculty of Medicine (Approval No. 2022/560; 16 December 2022). The institutional analysis used retrospectively collected clinical data from adult ICU patients receiving total parenteral nutrition (TPN). Written informed consent for participation was obtained from all patients or, where applicable, their legally authorized representatives before inclusion in the study. Clinical data were anonymized before analysis, and patient confidentiality and data privacy were maintained throughout the study.

## 3. Results

### 3.1. Study Population

A total of 844 patients from the institutional ICU cohort met the eligibility criteria and were included in the analysis. Based on the endpoint-defining APACHE-II assessment at T10, 214 patients (25.4%) met the predefined high-severity criterion (APACHE-II ≥ 20), whereas 630 (74.6%) remained below this threshold.

Baseline demographic and clinical characteristics are summarized in Table 3. Age, sex, BMI, chronic conditions, and baseline APACHE-II scores did not differ significantly between the two outcome groups (all *p* > 0.05). In particular, baseline APACHE-II scores were nearly identical (14.8 ± 9.5 vs. 14.7 ± 9.2; *p* = 0.926), indicating that the groups were not distinguishable by admission severity alone. As expected from the endpoint definition, final APACHE-II scores were substantially higher in the high-severity group (27.0 ± 5.5 vs. 8.4 ± 5.5; *p* < 0.001).

### 3.2. Longitudinal APACHE-II Patterns

**Figure 7:**
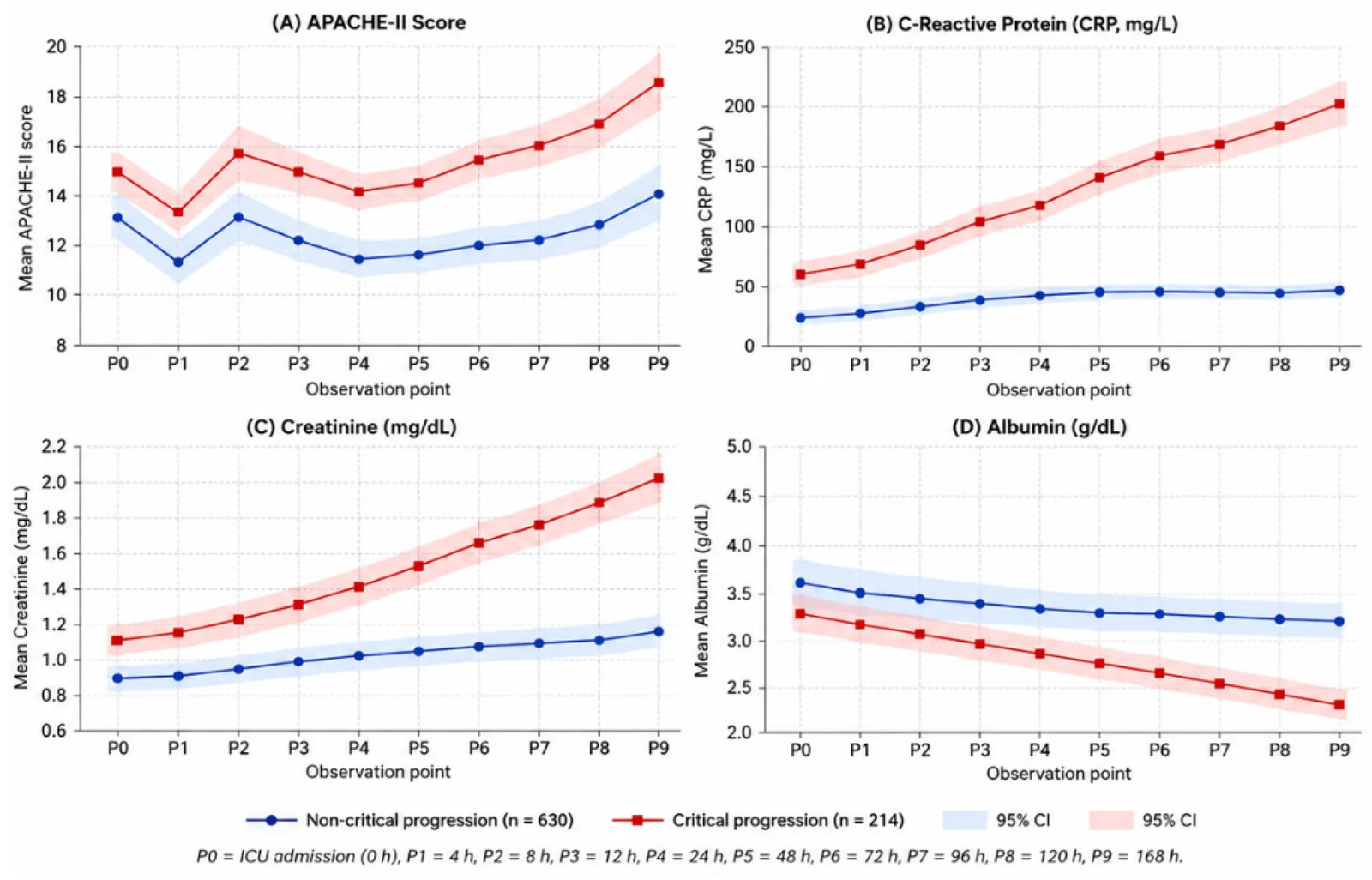
Mean longitudinal APACHE-II trajectories according to the subsequent high-severity endpoint. Mean APACHE-II scores across sequential predictor observations T0–T9 are shown for patients who did and did not meet the APACHE-II-defined high-severity endpoint at T10. Observation indices represent sequence order rather than standardized elapsed-time intervals; T10 is excluded from the plotted predictor trajectories.

Mean longitudinal APACHE-II trajectories across predictor observations T0–T9 are shown in Figure 7. The two outcome groups had comparable APACHE-II scores at T0 and exhibited substantial overlap and repeated crossings across subsequent observations. Thus, clear separation between groups was not apparent from the average trajectories alone.

Nevertheless, the observed within-sequence variation supported evaluation of repeated APACHE-II measurements and trajectory-derived descriptors using nonlinear machine-learning models. Their incremental predictive value relative to individual APACHE-II assessments and simpler trajectory summaries is examined in Section 3.3.

### 3.3. Machine Learning Performance and Incremental Value of Longitudinal APACHE-II

The predictive performance of the four primary feature representations is summarized in Table 4. Model A, combining the raw APACHE-II sequence T0–T9 with trajectory-derived descriptors, achieved the highest performance. CatBoost yielded a cross-validated ROC-AUC of 0.924 and an independent hold-out ROC-AUC of 0.921, with an accuracy of 0.840, sensitivity of 0.767, specificity of 0.865, precision of 0.660, and F1-score of 0.710.

An implementation review confirmed that feature construction, class weighting, out-of-fold probability generation, and training-derived threshold selection for these models were performed according to the specified analysis pipeline.

The longitudinal APACHE-II representation (Model A) achieved the strongest performance in the primary model-comparison analysis, with an out-of-fold ROC-AUC of 0.924 and an independent hold-out ROC-AUC of 0.921. Neither the addition of laboratory trajectories nor baseline characteristics improved discrimination. The admission-only and laboratory-only representations showed no useful discrimination under the optimized primary modeling pipeline and produced degenerate classifications at their training-derived operating thresholds. Because the primary endpoint was itself APACHE-II-defined, a separate incremental comparator analysis was subsequently performed using explicitly specified simplified APACHE-II representations to determine whether Model A’s performance could be reproduced by individual assessments, simple trajectory summaries, or conventional linear modeling (Table 5).

#### Incremental Comparator Analysis

Because the endpoint was defined using the subsequent APACHE-II assessment, additional analyses examined whether Model A’s performance could be explained by the latest available APACHE-II value or simple summaries of the preceding sequence. Results are presented in Table 5.

In the dedicated comparator analysis, admission APACHE-II (T0) alone achieved a hold-out ROC-AUC of 0.456, while T9 alone, change from baseline, and ordinal slope achieved ROC-AUCs of 0.553, 0.576, and 0.539, respectively. Logistic regression using the complete raw T0–T9 sequence achieved 0.610, and engineered trajectory descriptors achieved 0.633. All remained substantially below the full longitudinal CatBoost Model A (ROC-AUC = 0.921; AUPRC = 0.849), indicating that its discrimination was not reproduced by a single APACHE-II measurement, simple trajectory summaries, or linear modeling of the same longitudinal information.

### 3.4. Sequential Observation Sensitivity

As shown by the incremental analyses in Section 3.3, Model A substantially outperformed the latest APACHE-II assessment and simpler longitudinal summaries. To further examine dependence on observations closest to the endpoint, the APACHE-II predictor sequence was progressively truncated. Hold-out ROC-AUC values were 0.835 (T0–T3), 0.895 (T0–T4), 0.858 (T0–T5), 0.902 (T0–T6), 0.918 (T0–T7), 0.941 (T0–T8), and 0.921 (T0–T9). Thus, discrimination was retained after removal of later observations, although performance varied non-monotonically across truncation windows. Because reliable timestamps were unavailable, these results represent sequential-observation sensitivity rather than fixed clock-time prediction horizons. Complete discrimination, classification, calibration, threshold, and bootstrap results for all truncation windows are provided in Supplementary Table S4.

### 3.5. Internal Validation

**Figure 8:**
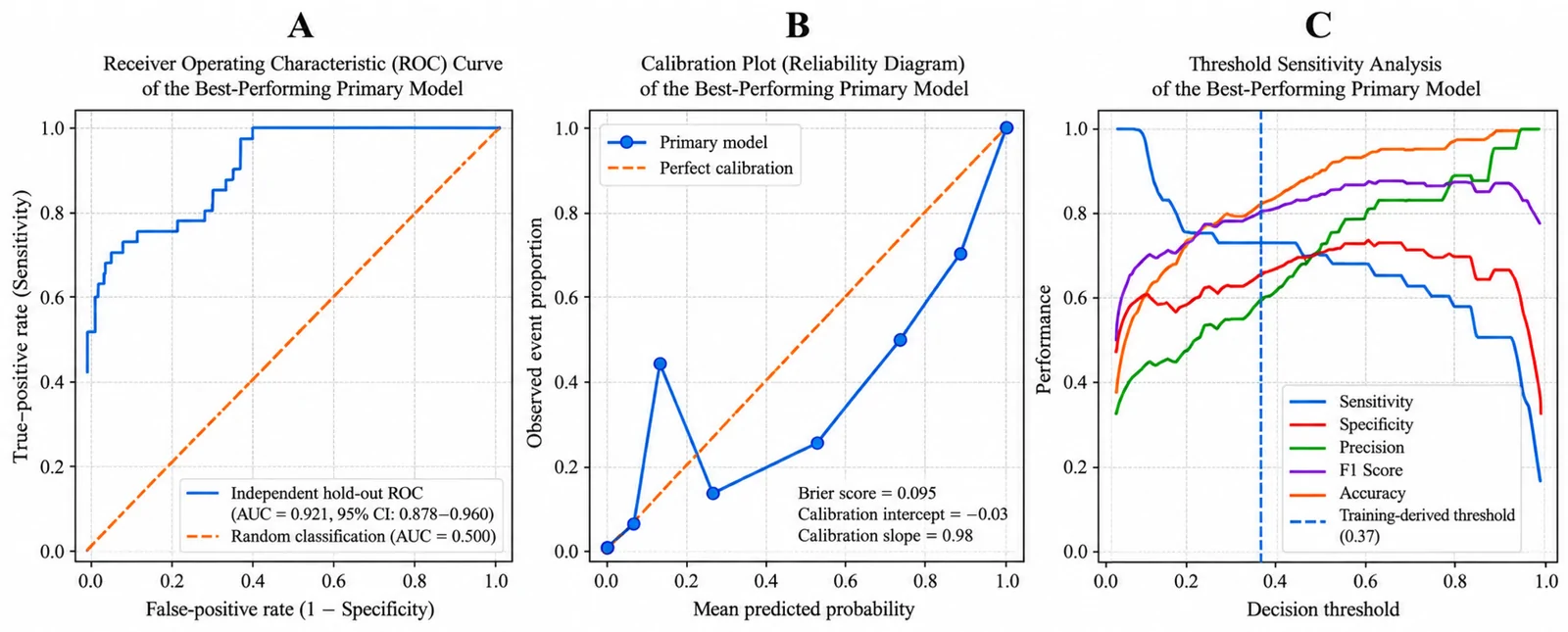
Internal validation of the longitudinal APACHE-II CatBoost model. (**A**) ROC curve for the independent hold-out cohort (ROC-AUC = 0.921; 95% CI: 0.878–0.960). (**B**) Calibration of predicted probabilities (Brier score = 0.095; intercept = − 0.03; slope = 0.98). (**C**) Threshold sensitivity across sensitivity, specificity, precision, F1-score, and accuracy. The dashed vertical line indicates the operating threshold of 0.37, selected from out-of-fold training predictions using the maximum Youden index.

The selected CatBoost Model A achieved a cross-validated ROC-AUC of 0.924 and an independent hold-out ROC-AUC of 0.921 (95% CI: 0.878–0.960). As shown in Figure 8A–C, hold-out calibration was good (Brier score = 0.095; calibration intercept = − 0.03; slope = 0.98), while threshold sensitivity analysis showed a balanced sensitivity–specificity trade-off around the training-derived operating threshold of 0.37.

The additional nested cross-validation analysis supported the stability of Model A, yielding a pooled out-of-fold ROC-AUC of 0.908 and a mean outer-fold ROC-AUC of 0.920 ± 0.038 (range: 0.857–0.955). These results were consistent with the original cross-validation and independent hold-out estimates.

### 3.6. Exploratory Cross-Cohort Portability Assessment

Because the eICU cohort did not contain an equivalent longitudinal APACHE-II representation or an endpoint corresponding to the institutional T10 APACHE-II ≥ 20 definition, the primary Model A could not undergo conventional external validation. Instead, a secondary XGBoost model restricted to variables that could be harmonized between the two cohorts was used for an exploratory assessment of cross-cohort portability.

**Figure 9:**
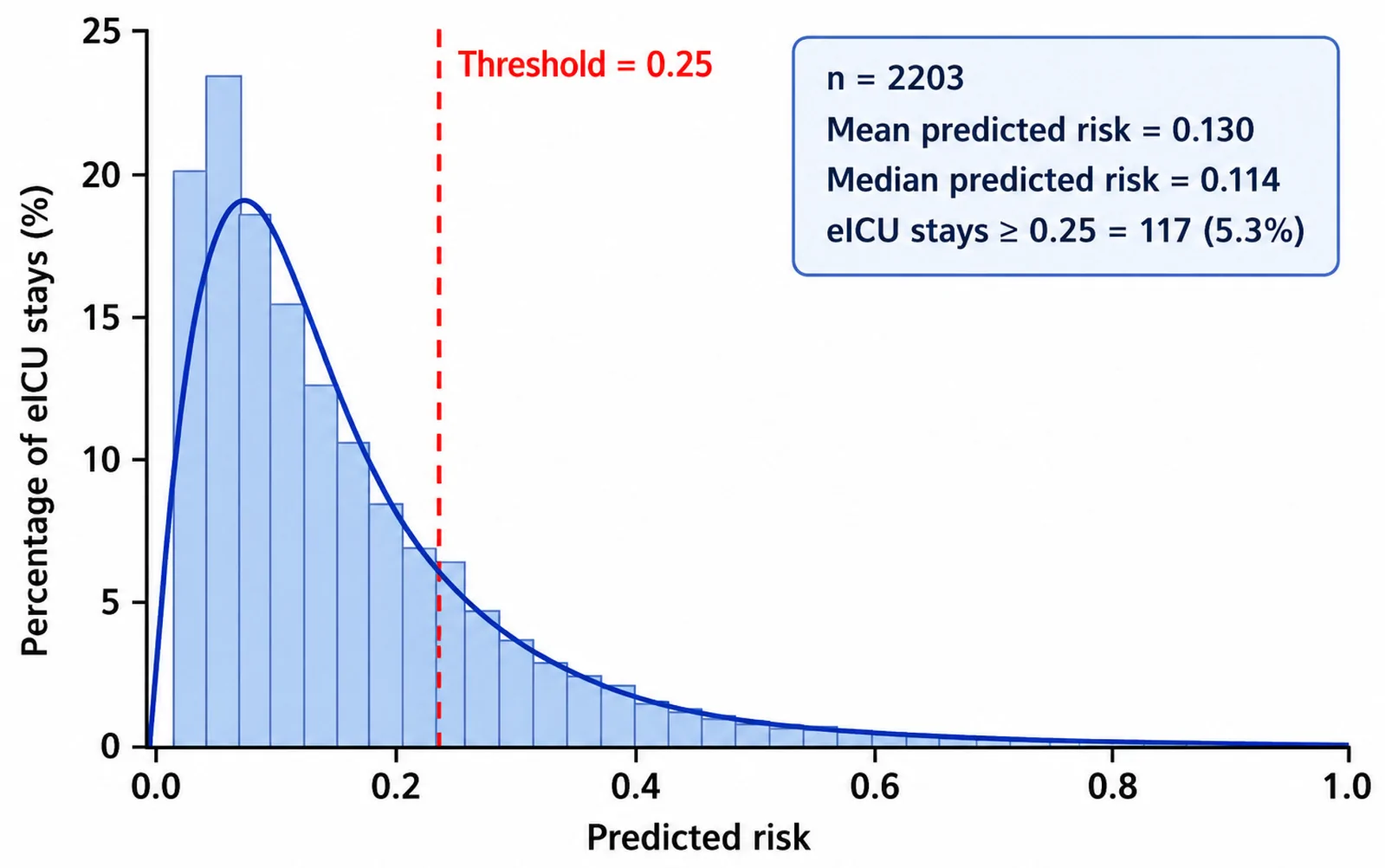
Exploratory cross-cohort portability assessment in the eICU cohort. Distribution of predicted risks generated by the secondary harmonized XGBoost model in 2203 eICU stays. The model was developed using variables shared between the institutional and eICU cohorts and applied to eICU without retraining or recalibration. The dashed vertical line indicates the institutional training-derived threshold of 0.25; 117 of 2203 eICU stays (5.3%) were above this threshold. The mean and median predicted risks were 0.130 and 0.114, respectively. Because eICU lacked an endpoint equivalent to the institutional T10 APACHE-II ≥ 20 definition, conventional external discrimination and calibration could not be estimated. Accordingly, this analysis represents exploratory cross-cohort portability assessment rather than conventional external validation.

Within the institutional cohort, this harmonized model showed limited discrimination, with an out-of-fold ROC-AUC of 0.512, a hold-out ROC-AUC of 0.523, and a hold-out AUPRC of 0.319. When subsequently applied to 2203 harmonized eICU stays, without retraining or recalibration on eICU data, the mean predicted risk was 0.130 and the median was 0.114. Using the institutional training-derived threshold of 0.25, 5.3% of eICU stays were classified as high risk (Figure 9).

Because an equivalent APACHE-II-defined endpoint was unavailable in eICU, conventional external discrimination and calibration of the primary study outcome could not be estimated. Accordingly, the eICU analysis was interpreted as an exploratory assessment of feature compatibility and predicted-risk transport rather than external validation. The observed predicted-risk distribution illustrates the challenges of transporting the harmonized representation across heterogeneous cohorts and emphasizes the need for future validation in datasets containing equivalent longitudinal APACHE-II measurements, endpoint definitions, and observation timing.

### 3.7. Model Explainability

**Figure 10:**
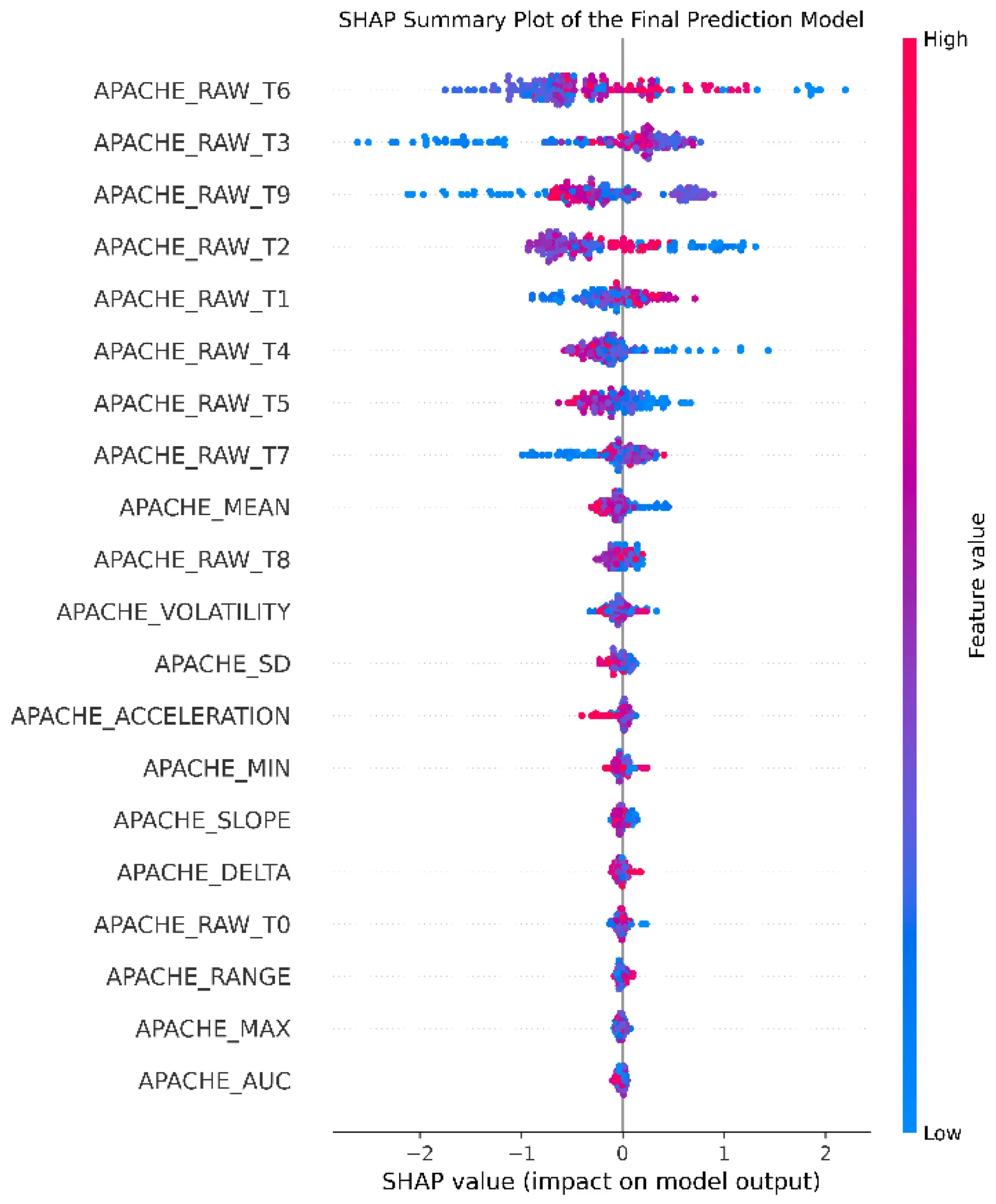
SHAP summary plot for the final longitudinal APACHE-II CatBoost model. Features are ranked according to their contribution to model predictions. Individual APACHE-II observations, particularly T6, T3, T9, T2, and T1, showed prominent contributions, while trajectory-derived descriptors provided complementary information. SHAP values represent model-level associations with predictions and do not establish causal or independent clinical effects.

SHAP analysis was applied to the final CatBoost Model A to quantify the contribution of individual features to model predictions. The resulting feature-contribution distribution is shown in Figure 10.

Individual APACHE-II observations contributed most strongly to the model predictions, with T6, T3, T9, T2, and T1 among the highest-ranked features. Trajectory-derived descriptors, including summary level, variability, change, slope, and acceleration measures, provided additional contributions but generally ranked below the individual APACHE-II observations. These findings indicate that predictions from Model A were based on information distributed across multiple observations in the longitudinal sequence rather than being dominated solely by the latest available APACHE-II value (T9).

Because Model A contained only longitudinal APACHE-II measurements and their derived descriptors, laboratory and baseline demographic variables were not included in its SHAP feature space. Importantly, SHAP values quantify feature contributions to the fitted model predictions and should not be interpreted as evidence of causal effects or independent clinical importance.

## 4. Discussion

This study evaluated an explainable machine learning framework for predicting a subsequent APACHE-II-defined high-severity state from repeated APACHE-II assessments in ICU patients receiving TPN. The longitudinal APACHE-II representation provided the strongest internal discrimination, substantially outperforming admission APACHE-II, the latest available assessment, simple trajectory summaries, and linear models using the same longitudinal information. Sequential observation-truncation and nested cross-validation analyses further supported the robustness of the primary findings. However, the shared APACHE-II construct between predictors and endpoint, the absence of standardized observation timing, and the limited portability of a secondary harmonized representation are important considerations when interpreting these results.

### 4.1. Principal Findings

The principal finding was that nonlinear modeling of repeated APACHE-II assessments provided substantially greater discrimination of a subsequent APACHE-II-defined high-severity state than simpler severity representations. Model A outperformed admission APACHE-II, the latest assessment (T9), change from baseline, ordinal slope, and logistic models using the same longitudinal information. Sequential observation truncation further showed that discrimination was retained after later observations were removed, although the observation indices cannot be interpreted as fixed clock-time prediction horizons.

The CatBoost model showed strong internal discrimination and good calibration, with nested cross-validation supporting the stability of the primary findings. SHAP analysis indicated that multiple APACHE-II observations contributed to model predictions, while trajectory-derived descriptors provided complementary information. These contributions represent model-level associations and should not be interpreted as causal or independent clinical effects. Adding laboratory trajectories and baseline characteristics did not improve performance over the APACHE-II-only representation for this endpoint.

Importantly, the primary Model A could not undergo conventional external validation in eICU because equivalent longitudinal APACHE-II measurements and an equivalent endpoint were unavailable. The secondary harmonized analysis therefore represents exploratory portability assessment only. Overall, the findings demonstrate strong internal prediction of a subsequent APACHE-II-defined high-severity state, but interpretation should account for the shared APACHE-II construct between predictors and endpoint and should not be extended to independent clinical deterioration without further validation.

### 4.2. Value of Longitudinal Severity Assessment

Critical illness is dynamic, and a single admission assessment may not fully represent subsequent changes in patient severity. In this study, repeated APACHE-II assessments provided substantially greater predictive information than admission APACHE-II alone. Importantly, the incremental analyses showed that this advantage was not reproduced by the latest assessment, simple change from baseline, ordinal slope, or linear modeling of the longitudinal sequence, supporting the value of integrating information distributed across repeated observations.

These findings support the use of longitudinal severity representations in critical care prediction while requiring cautious interpretation. Because T0–T9 were sequential observation indices rather than standardized time intervals, the results do not establish a specific early-warning horizon. Moreover, the APACHE-II-defined endpoint shares its underlying construct with the predictor sequence; therefore, the findings demonstrate prediction of a subsequent high-severity state rather than independent clinical deterioration. Future studies incorporating standardized timestamps and independent outcomes such as mortality, organ-support requirements, or clinically adjudicated deterioration are needed to determine the broader prognostic value of this approach.

### 4.3. Comparison with Previous Studies

Machine learning has increasingly been applied to outcome prediction in critical care, including mortality, prolonged ICU stay, and organ failure. Conventional severity scores such as APACHE-II, SOFA, and SAPS are widely used for risk assessment, while more recent machine learning approaches incorporate broader clinical and laboratory information [7,8,9]. Growing evidence also suggests that repeated clinical measurements can provide predictive information beyond static baseline assessments [15,16,17,18]. The present findings extend this work by showing that nonlinear modeling of repeated APACHE-II assessments provided substantially greater discrimination of a subsequent APACHE-II-defined high-severity state than admission severity, the latest assessment, simple trajectory summaries, or linear modeling of the same longitudinal information.

Explainability is also increasingly emphasized in clinical machine learning because complex models can be difficult to interpret [42,43,44,55]. In the present study, SHAP analysis identified multiple APACHE-II observations and trajectory-derived descriptors that contributed to CatBoost predictions. This provides model-level insight into how the longitudinal representation was used, although SHAP contributions should not be interpreted as causal effects or independent clinical importance.

The study also highlights challenges in cross-cohort evaluation. Although internal performance was supported by independent hold-out testing and nested cross-validation, conventional external validation of the primary model was not possible in eICU because equivalent longitudinal APACHE-II measurements and an equivalent endpoint were unavailable. The limited performance of the secondary harmonized representation illustrates the difficulty of transferring models when variable definitions and outcome structures differ across datasets [47,48]. Future multicenter studies using harmonized longitudinal measurements, standardized timing, and independently defined clinical outcomes will therefore be necessary to establish broader generalizability.

### 4.4. Clinical Implications

Repeated assessment of illness severity may provide a more informative basis for risk stratification than reliance on admission severity alone. In this cohort, nonlinear modeling of longitudinal APACHE-II information identified patients who subsequently met the APACHE-II-defined high-severity threshold with strong internal discrimination. The findings therefore support further investigation of longitudinal severity modeling as a potential component of dynamic ICU risk assessment, rather than establishing its readiness for clinical decision-making.

The study population consisted specifically of ICU patients receiving total parenteral nutrition, a clinically complex group requiring close physiological and metabolic monitoring. Accordingly, the findings are most directly applicable to this population and should not be assumed to generalize to the broader ICU population. Before clinical implementation, the framework requires prospective multicenter validation using standardized observation timing and, importantly, independent clinically meaningful outcomes such as mortality, organ-support requirements, or adjudicated deterioration.

### 4.5. Model Interpretability and Explainable AI

Explainability is important in clinical machine learning because complex models may otherwise provide limited insight into how predictions are generated. In this study, SHAP analysis quantified the contribution of individual APACHE-II observations and trajectory-derived descriptors to predictions from the final CatBoost model.

Multiple APACHE-II observations contributed prominently to model predictions, while trajectory-derived descriptors provided complementary information. This indicates that the fitted model used information distributed across the longitudinal representation rather than relying exclusively on a single APACHE-II assessment. However, SHAP values describe associations within the fitted model and should not be interpreted as evidence of causality, independent clinical importance, or physiological mechanisms.

### 4.6. Cross-Cohort Portability

The secondary harmonized model showed limited discrimination within the institutional cohort, indicating that the shared-variable representation did not preserve the predictive information available to the primary longitudinal APACHE-II model. Therefore, its weak portability should not be interpreted as external failure of Model A, which could not be directly evaluated in eICU because equivalent longitudinal APACHE-II measurements were unavailable.

When applied to eICU, the harmonized model produced a different predicted-risk distribution, with 5.3% of stays exceeding the institutional training-derived threshold. However, because eICU lacked an endpoint equivalent to the institutional T10 APACHE-II ≥ 20 definition, conventional external discrimination and calibration could not be assessed. The analysis therefore illustrates the challenges of cross-cohort feature harmonization and risk transport when measurement structures and endpoint definitions differ, rather than providing conventional external validation.

### 4.7. Strengths and Limitations

This study has several strengths. First, it evaluated repeated APACHE-II assessments using a leakage-controlled framework that separated predictor observations (T0–T9) from the endpoint-defining assessment (T10). Second, the primary findings were evaluated using five-fold cross-validation, an independent hold-out cohort, nested cross-validation, and sequential observation-truncation analyses. Third, the incremental comparator analysis directly assessed whether the performance of the longitudinal CatBoost model could be reproduced using admission APACHE-II, the latest available assessment, simple trajectory descriptors, or conventional logistic regression. Finally, SHAP analysis provided model-level information regarding the contribution of individual APACHE-II observations and trajectory-derived features to the predictions.

Several limitations should nevertheless be considered. Most importantly, the predictors and endpoint were derived from the same APACHE-II construct. Although T10 was excluded from the predictor set, the strong discrimination of Model A should therefore be interpreted as prediction of a subsequent APACHE-II-defined high-severity state rather than prediction of an independent clinical outcome. In addition, T0–T10 represented sequential observation indices rather than standardized clock-time intervals because reliable timestamps were unavailable. Consequently, fixed prediction horizons such as 24, 48, or 72 h could not be evaluated. The retrospective single-center institutional cohort was also restricted to ICU patients receiving TPN, which limits generalizability to broader critical-care populations. Finally, conventional external validation of the primary Model A was not possible because eICU lacked both an equivalent longitudinal APACHE-II representation and an endpoint corresponding to the institutional T10 APACHE-II ≥ 20 definition. The eICU analysis should therefore be interpreted only as an exploratory assessment of cross-cohort feature compatibility and predicted-risk portability.

### 4.8. Future Directions

Future studies should prospectively evaluate the framework in larger, multicenter ICU cohorts using standardized and precisely timestamped observation intervals. Such studies should define clinically meaningful prediction horizons and evaluate independent outcomes, including mortality, organ-support requirements, ICU length of stay, or other prospectively specified deterioration endpoints, to determine whether longitudinal APACHE-II modeling provides prognostic value beyond prediction of a subsequent APACHE-II-defined severity state. External validation should also be performed in cohorts containing equivalent predictor definitions, observation timing, and outcome ascertainment.

Further methodological work should investigate whether integration of additional longitudinal physiological, laboratory, treatment, and nutritional information improves performance beyond repeated APACHE-II assessments. Evaluation of alternative longitudinal modeling approaches and prospective assessment of calibration, decision-curve utility, and clinically appropriate operating thresholds would further clarify the potential value of the framework. Because the present cohort consisted specifically of patients receiving TPN, future studies should also examine performance in broader ICU populations and determine whether TPN-related clinical characteristics modify model behavior.

## 5. Conclusions

This study demonstrated that nonlinear modeling of repeated APACHE-II assessments can provide strong internal discrimination of a subsequent APACHE-II-defined high-severity state in ICU patients receiving TPN. The longitudinal CatBoost model outperformed admission APACHE-II, the latest available assessment, simple trajectory summaries, and linear models using the same longitudinal information, while nested cross-validation and sequential observation-truncation analyses supported the robustness of the primary findings. However, the shared APACHE-II construct between predictors and endpoint, the absence of standardized observation timing, and the inability to externally validate the primary model using an equivalent eICU representation limit broader interpretation. Future prospective multicenter studies should evaluate standardized prediction horizons and independent clinical outcomes to determine whether longitudinal APACHE-II modeling provides clinically useful prognostic information beyond prediction of a subsequent APACHE-II-defined severity state.

## Figures and Tables

**Table 1:** Characteristics and analytic roles of the institutional and eICU cohorts.

Characteristic	Institutional Cohort	eICU Cohort
Initial cohort described	844 patients	2520 ICU records/stays
Final sample used in primary/portability analysis	844 patients	2203 harmonized ICU stays
Study design	Retrospective, single-center	Retrospective, multicenter
Data source	Institutional ICU database	eICU Collaborative Research Database Demonstration
Country	Türkiye	United States
Number of participating hospitals	1	Multiple hospitals
Mean age (years)	61.4 ± 16.6	62.8 ± 16.9 *
Male sex, *n* (%)	681 (80.7%)	1407 (55.8%) *
Mean BMI (kg/m^2^)	25.8 ± 12.7	27.3 ± 7.2 *
Equivalent longitudinal APACHE-II assessments	Available	Not available
Longitudinal laboratory measurements	Available	Available for harmonized variables
Institutional study endpoint	T10 APACHE-II ≥ 20	No equivalent endpoint available
Primary Model A applicable	Yes	No
Model used for portability assessment	—	Secondary harmonized XGBoost model
Model retraining/recalibration using eICU	—	No
Role in this study	Model development and internal validation	Exploratory cross-cohort portability assessment

Note: BMI, body mass index; ICU, intensive care unit; APACHE-II, Acute Physiology and Chronic Health Evaluation II. * Descriptive characteristics refer to the initially described eICU cohort (*n* = 2520), whereas the final harmonized subset used for the exploratory portability analysis comprised 2203 ICU stays.

**Table 2:** Summary of the primary feature representations evaluated in the study and their respective analytical purposes.

Feature Representation	Purpose
Clinical baseline	Single-assessment comparator
Model A	Longitudinal APACHE-II information
Model B	Laboratory information independent of APACHE-II
Model C	Complementary APACHE-II and laboratory information
Model D	Added value of static patient characteristics

**Table 3:** Baseline demographic and clinical characteristics of the institutional cohort according to the APACHE-II-defined high-severity endpoint.

Variable	Overall (*n* = 844)	Below High-Severity Threshold (*n* = 630)	High-Severity State (*n* = 214)	*p*-Value
Age (years)	61.4 ± 16.6	61.9 ± 16.1	60.1 ± 17.8	0.202
Male sex, *n* (%)	681 (80.7%)	510 (81.0%)	171 (79.9%)	0.814
BMI (kg/m^2^)	25.8 ± 12.7	25.8 ± 12.2	25.9 ± 14.0	0.856
Diabetes mellitus, *n* (%)	326 (38.6%)	242 (38.4%)	84 (39.3%)	0.891
Hypertension, *n* (%)	448 (53.1%)	335 (53.2%)	113 (52.8%)	0.988
Coronary artery disease, *n* (%)	81 (9.6%)	56 (8.9%)	25 (11.7%)	0.287
Chronic kidney disease, *n* (%)	64 (7.6%)	49 (7.8%)	15 (7.0%)	0.828
Chronic obstructive pulmonary disease, *n* (%)	70 (8.3%)	54 (8.6%)	16 (7.5%)	0.720
Hepatitis, *n* (%)	52 (6.2%)	36 (5.7%)	16 (7.5%)	0.446
Baseline APACHE-II score	14.7 ± 9.3	14.7 ± 9.2	14.8 ± 9.5	0.926
Final APACHE-II score	13.1 ± 9.9	8.4 ± 5.5	27.0 ± 5.5	<0.001

Values are presented as mean ± standard deviation for continuous variables and *n* (%) for categorical variables. Continuous variables were compared using Welch’s independent-samples *t*-test and categorical variables using the χ^2^ test. The high-severity endpoint was defined as APACHE-II at T10 ≥ 20.

**Table 4:** Performance of the best-performing algorithm for each primary feature representation.

Feature Representation	Best Algorithm	OOF CV ROC-AUC	Hold-Out ROC-AUC	Accuracy	Sensitivity	Specificity	Precision	F1-Score
Admission APACHE-II only	Logistic Regression	0.500	0.500	0.254	1.000	0.000	0.254	0.406
Model A: Longitudinal APACHE-II	CatBoost	0.924	0.921	0.840	0.767	0.865	0.660	0.710
Model B: Laboratory trajectories	Logistic Regression	0.500	0.500	0.254	1.000	0.000	0.254	0.406
Model C: APACHE-II + laboratory	CatBoost	0.893	0.891	0.834	0.721	0.873	0.660	0.689
Model D: Full multimodal representation	CatBoost	0.895	0.901	0.822	0.791	0.833	0.618	0.694

Note: For each feature representation, the algorithm with the highest out-of-fold training ROC-AUC was selected before independent hold-out evaluation. Classification thresholds were derived exclusively from out-of-fold training predictions using the maximum Youden index. The admission-only and laboratory-only models produced non-informative probability estimates under the optimized primary modeling pipeline and degenerate hold-out classifications at their training-derived thresholds.

**Table 5:** Incremental comparison of Model A with simpler APACHE-II representations on the independent hold-out cohort.

Predictor Representation	Model	Hold-Out ROC-AUC	Hold-Out AUPRC
Admission APACHE-II (T0)	Logistic Regression	0.456	0.240
Latest APACHE-II (T9)	Logistic Regression	0.553	0.267
Change from baseline (T9−T0)	Logistic Regression	0.576	0.289
Ordinal trajectory slope	Logistic Regression	0.539	0.323
T9 + change + ordinal slope	Logistic Regression	0.552	0.286
Raw APACHE-II sequence T0–T9	Logistic Regression	0.610	0.385
Engineered APACHE-II descriptors	Logistic Regression	0.633	0.389
Raw T0–T9 + engineered descriptors	Logistic Regression	0.610	0.382
Full longitudinal Model A	CatBoost	0.921	0.849

## Data Availability

The institutional dataset contains sensitive patient information and cannot be publicly released because of ethical and institutional restrictions. De-identified data supporting the findings of this study may be made available from the corresponding author upon reasonable request and subject to institutional approval. The eICU Collaborative Research Database Demonstration dataset is publicly available to researchers.

## Supplementary Materials

The following supporting information can be downloaded at: https://www.mdpi.com/article/10.3390/diagnostics16193118/s1, Table S1: Complete performance of all machine-learning algorithms evaluated across the primary feature representations; Table S2: Feature-level missingness audit for longitudinal APACHE-II and laboratory variables in the institutional cohort (*n* = 844); Table S3: Nested cross-validation results for Model A; Table S4: Sequential observation-truncation sensitivity analysis for longitudinal APACHE-II Model A; Figure S1: Institutional cohort flow and data partitioning.
