# Supplementary material for: Explainable Machine Learning for Prediction of Future High-Severity States Using Longitudinal APACHE-II Trajectories: Internal Validation and Cross-Cohort Portability Assessment
Source: Diagnostics (Basel). 2026 Sep 25;16(19):3118. doi: 10.3390/diagnostics16193118 (PMC13664044; doi:10.3390/diagnostics16193118)
Supplement: Supplementary file 1 [file diagnostics-16-03118-s001.zip › diagnostics-4481182-supplementary.pdf]

Supplementary Table S1. Complete performance of all machine-learning algorithms evaluated across the primary feature representations

Feature representation	Algorithm	OOF CV ROC-AUC	Hold-out ROC-AUC	Hold-out AUPRC	Threshold	Accuracy	Sensitivity	Specificity	Precision	F1-score	Brier score
Clinical baseline: Admission APACHE-II (T0)	Logistic Regression	0.500	0.500	0.254	0.050	0.254	1.000	0.000	0.254	0.406	0.250
Clinical baseline: Admission APACHE-II (T0)	Random Forest	0.468	0.557	0.287	0.050	0.278	1.000	0.032	0.261	0.413	0.253
Clinical baseline: Admission APACHE-II (T0)	Extra Trees	0.472	0.573	0.295	0.260	0.278	1.000	0.032	0.261	0.413	0.252
Clinical baseline: Admission APACHE-II (T0)	XGBoost	0.497	0.556	0.278	0.550	0.604	0.326	0.698	0.269	0.295	0.249
Clinical baseline: Admission APACHE-II (T0)	LightGBM	0.495	0.568	0.279	0.500	0.562	0.488	0.587	0.288	0.362	0.249
Clinical baseline: Admission APACHE-II (T0)	CatBoost	0.489	0.532	0.257	0.490	0.556	0.674	0.516	0.322	0.436	0.251
**Model A: Longitudinal APACHE-II**	Logistic Regression	0.613	0.611	0.383	0.480	0.538	0.535	0.540	0.284	0.371	0.237
**Model A: Longitudinal APACHE-II**	Random Forest	0.916	0.920	**0.871**	0.420	**0.870**	0.767	**0.905**	**0.733**	**0.750**	**0.093**
**Model A: Longitudinal APACHE-II**	Extra Trees	0.895	0.911	0.855	0.430	0.858	0.814	0.873	0.686	0.745	0.114
**Model A: Longitudinal APACHE-II**	XGBoost	0.918	0.919	0.847	0.380	0.834	**0.814**	0.841	0.636	0.714	0.102
**Model A: Longitudinal APACHE-II**	LightGBM	0.922	0.910	0.842	0.340	0.817	0.767	0.833	0.611	0.680	0.105
**Model A: Longitudinal APACHE-II**	**CatBoost**	**0.924**	**0.921**	0.849	0.370	0.840	0.767	0.865	0.660	0.710	0.096
Model B: Laboratory trajectories	Logistic Regression	0.500	0.500	0.254	0.050	0.254	1.000	0.000	0.254	0.406	0.250
Model B: Laboratory trajectories	Random Forest	0.492	0.512	0.309	0.420	0.675	0.163	0.849	0.269	0.203	0.203

Feature representation	Algorithm	OOF CV ROC-AUC	Hold-out ROC-AUC	Hold-out AUPRC	Threshold	Accuracy	Sensitivity	Specificity	Precision	F1-score	Brier score
Model B: Laboratory trajectories	Extra Trees	0.486	0.552	0.332	0.320	0.414	0.744	0.302	0.267	0.393	0.203
Model B: Laboratory trajectories	XGBoost	0.495	0.528	0.301	0.120	0.391	0.791	0.254	0.266	0.398	0.216
Model B: Laboratory trajectories	LightGBM	0.497	0.543	0.301	0.380	0.645	0.209	0.794	0.257	0.231	0.232
Model B: Laboratory trajectories	CatBoost	0.488	0.531	0.298	0.500	0.704	0.163	0.889	0.333	0.219	0.241
Model C: APACHE-II + laboratory	Logistic Regression	0.518	0.561	0.307	0.490	0.444	0.628	0.381	0.257	0.365	0.245
Model C: APACHE-II + laboratory	Random Forest	0.819	0.884	0.759	0.260	0.769	0.837	0.746	0.529	0.649	0.125
Model C: APACHE-II + laboratory	Extra Trees	0.794	0.865	0.762	0.440	0.834	0.744	0.865	0.653	0.696	0.156
Model C: APACHE-II + laboratory	XGBoost	0.849	0.873	0.779	0.360	0.811	0.721	0.841	0.608	0.660	0.112
Model C: APACHE-II + laboratory	LightGBM	0.859	**0.892**	**0.813**	0.300	0.822	**0.791**	0.833	0.618	**0.694**	**0.103**
Model C: APACHE-II + laboratory	**CatBoost**	**0.893**	0.891	0.798	0.360	**0.834**	0.721	**0.873**	**0.660**	0.689	0.107
Model D: Full multimodal representation	Logistic Regression	0.529	0.557	0.317	0.480	0.408	0.698	0.310	0.256	0.375	0.244
Model D: Full multimodal representation	Random Forest	0.820	0.875	0.752	0.260	0.769	**0.837**	0.746	0.529	0.649	0.128
Model D: Full multimodal representation	Extra Trees	0.787	0.864	0.766	0.430	**0.834**	0.767	0.857	0.647	**0.702**	0.156
Model D: Full multimodal representation	XGBoost	0.843	0.892	0.791	0.380	0.828	0.698	**0.873**	**0.652**	0.674	0.110
Model D: Full multimodal representation	LightGBM	0.856	0.895	0.804	0.250	0.746	0.791	0.730	0.500	0.613	0.105

Feature representation	Algorithm	OOF CV ROC-AUC	Hold-out ROC-AUC	Hold-out AUPRC	Threshold	Accuracy	Sensitivity	Specificity	Precision	F1-score	Brier score
Model D: Full multimodal representation	**CatBoost**	**0.895**	**0.901**	**0.812**	0.280	0.822	0.791	0.833	0.618	0.694	**0.103**

Note: OOF CV ROC-AUC denotes the area under the receiver operating characteristic curve calculated from out-of-fold predictions in the institutional training/development cohort (n = 675). Hold-out metrics were calculated in the independent institutional hold-out cohort (n = 169). Classification metrics were calculated using the operating threshold selected exclusively from out-of-fold training predictions by maximizing the Youden index. AUPRC, area under the precision–recall curve; OOF, out-of-fold; ROC-AUC, area under the receiver operating characteristic curve.

Supplementary Table S2. Feature-level missingness audit for longitudinal APACHE-II and laboratory variables in the institutional cohort (n = 844)

Variable	T0	T1	T2	T3	T4	T5	T6	T7	T8	T9
**APACHE-II**	0 (0.00)	0 (0.00)	0 (0.00)	0 (0.00)	0 (0.00)	0 (0.00)	0 (0.00)	0 (0.00)	0 (0.00)	0 (0.00)
Albumin	0 (0.00)	0 (0.00)	0 (0.00)	0 (0.00)	0 (0.00)	0 (0.00)	0 (0.00)	0 (0.00)	0 (0.00)	0 (0.00)
Alkaline phosphatase (ALP)	0 (0.00)	3 (0.36)	1 (0.12)	2 (0.24)	5 (0.59)	2 (0.24)	0 (0.00)	2 (0.24)	0 (0.00)	3 (0.36)
Alanine aminotransferase (ALT)	2 (0.24)	2 (0.24)	1 (0.12)	0 (0.00)	**13 (1.54)**	0 (0.00)	1 (0.12)	0 (0.00)	0 (0.00)	0 (0.00)
Aspartate aminotransferase (AST)	0 (0.00)	0 (0.00)	0 (0.00)	0 (0.00)	0 (0.00)	0 (0.00)	0 (0.00)	0 (0.00)	0 (0.00)	0 (0.00)
Calcium	0 (0.00)	0 (0.00)	0 (0.00)	0 (0.00)	0 (0.00)	0 (0.00)	0 (0.00)	0 (0.00)	0 (0.00)	0 (0.00)
Creatinine	0 (0.00)	0 (0.00)	0 (0.00)	0 (0.00)	0 (0.00)	1 (0.12)	0 (0.00)	0 (0.00)	0 (0.00)	0 (0.00)
C-reactive protein (CRP)	0 (0.00)	0 (0.00)	2 (0.24)	3 (0.36)	3 (0.36)	3 (0.36)	3 (0.36)	2 (0.24)	2 (0.24)	1 (0.12)
Gamma-glutamyl transferase (GGT)	0 (0.00)	0 (0.00)	0 (0.00)	0 (0.00)	0 (0.00)	0 (0.00)	0 (0.00)	0 (0.00)	4 (0.47)	0 (0.00)

Variable	T0	T1	T2	T3	T4	T5	T6	T7	T8	T9
Glucose	0 (0.00)	0 (0.00)	0 (0.00)	0 (0.00)	0 (0.00)	0 (0.00)	0 (0.00)	0 (0.00)	0 (0.00)	0 (0.00)
Magnesium	0 (0.00)	0 (0.00)	0 (0.00)	0 (0.00)	0 (0.00)	0 (0.00)	0 (0.00)	0 (0.00)	0 (0.00)	0 (0.00)
Potassium	0 (0.00)	0 (0.00)	0 (0.00)	0 (0.00)	0 (0.00)	0 (0.00)	0 (0.00)	0 (0.00)	0 (0.00)	0 (0.00)
Total bilirubin	2 (0.24)	1 (0.12)	2 (0.24)	0 (0.00)	1 (0.12)	0 (0.00)	4 (0.47)	4 (0.47)	6 (0.71)	4 (0.47)

Note: Missingness was assessed at the individual observation level before model-specific imputation. APACHE-II predictor observations T0–T9 were complete for all 844 patients. The T10 APACHE-II assessment was retained exclusively for endpoint definition and was not used as a predictor. Laboratory missingness was low overall, with the highest feature-level missingness observed for ALT at T4 (13/844, 1.54%). Missing laboratory values were handled using median values estimated exclusively from the institutional training data and subsequently applied to validation and hold-out data to prevent information leakage. Derived trajectory features contained no missing values after feature construction and preprocessing.

Supplementary Table S3. Nested cross-validation results for Model A

Outer fold	Outer training, n	Outer validation, n	Best inner-CV ROC-AUC	Outer validation ROC-AUC	Outer validation AUPRC	Selected CatBoost parameters
1	540	135	0.895	0.942	0.873	iterations = 500; learning rate = 0.01; depth = 6; L2 leaf reg. = 3; random strength = 0.0; border count = 128
2	540	135	0.897	0.927	0.878	iterations = 200; learning rate = 0.03; depth = 6; L2 leaf reg. = 3; random strength = 0.0; border count = 64
3	540	135	0.922	0.857	0.743	iterations = 300; learning rate = 0.10; depth = 8; L2 leaf reg. = 7; random strength = 2.0; border count = 128
4	540	135	0.874	0.955	0.895	iterations = 200; learning rate = 0.05; depth = 8; L2 leaf reg. = 7; random strength = 0.0; border count = 64
5	540	135	0.892	0.920	0.850	iterations = 200; learning rate = 0.10; depth = 6; L2 leaf reg. = 10; random strength = 2.0; border count = 128
**Overall**	—	—	—	**0.920 ± 0.038**	—	—
**Pooled nested OOF**	**675**	—	—	**0.908**	**0.826**	—

Note: Nested cross-validation was performed only within the institutional training/development cohort (n = 675) as an additional robustness analysis. Five outer stratified folds were used for performance estimation. Within each outer-training fold, hyperparameter optimization was performed using three-fold inner cross-validation with 20 randomized-search iterations and ROC-AUC as the optimization criterion. The pooled nested out-of-fold ROC-AUC was 0.908 and pooled AUPRC was 0.826. The mean outer-fold ROC-AUC was 0.920 ± 0.038, with a range of 0.857–0.955. The independent hold-out cohort was not involved in nested cross-validation.

Supplementary Table S4. Sequential observation-truncation sensitivity analysis for longitudinal APACHE-II Model A

Available APACHE-II window	Raw observations, n	Features, n	OOF training ROC-AUC	Training-derived threshold	Hold-out ROC-AUC	Hold-out AUPRC	Accuracy	Sensitivity	Specificity	Precision	F1-score	Brier score	ROC-AUC 95% CI
T0–T3	4	16	0.805	0.200	0.835	0.709	0.704	0.860	0.651	0.457	0.597	0.133	0.753–0.906
T0–T4	5	17	0.859	0.318	0.895	0.774	0.817	0.791	0.825	0.607	0.687	0.111	0.842–0.943
T0–T5	6	18	0.869	0.292	0.858	0.736	0.775	0.814	0.762	0.538	0.648	0.126	0.789–0.917
T0–T6	7	19	0.913	0.460	0.902	0.842	0.858	0.744	0.897	0.711	0.727	0.096	0.838–0.957
T0–T7	8	20	0.920	0.351	0.918	0.858	0.870	0.744	0.913	0.744	0.744	0.090	0.867–0.962
T0–T8	9	21	0.916	0.398	**0.941**	**0.886**	**0.876**	0.814	0.897	0.729	**0.769**	**0.082**	0.894–0.976
T0–T9	10	22	**0.924**	0.399	0.921	0.849	0.852	0.767	0.881	0.688	0.725	0.096	0.877–0.960

Note: Sequential observation truncation was performed to assess whether Model A depended disproportionately on APACHE-II observations closest to the endpoint. For each truncated sequence, the available raw APACHE-II observations and corresponding trajectory descriptors were reconstructed, and the model was evaluated using the same institutional training/hold-out framework. Classification thresholds were derived from out-of-fold training predictions and fixed before hold-out evaluation. Because reliable elapsed-time information between observations was unavailable, T0–T3 through T0–T9 represent progressively longer ordered observation sequences rather than fixed clinical time horizons. T10 was excluded from all predictor sets and retained exclusively for endpoint definition.

Supplementary Figure S1 summarizes the institutional cohort-selection and data-partitioning process. All 844 records in the curated institutional dataset satisfied the analytical eligibility and data-quality requirements and were retained for analysis.

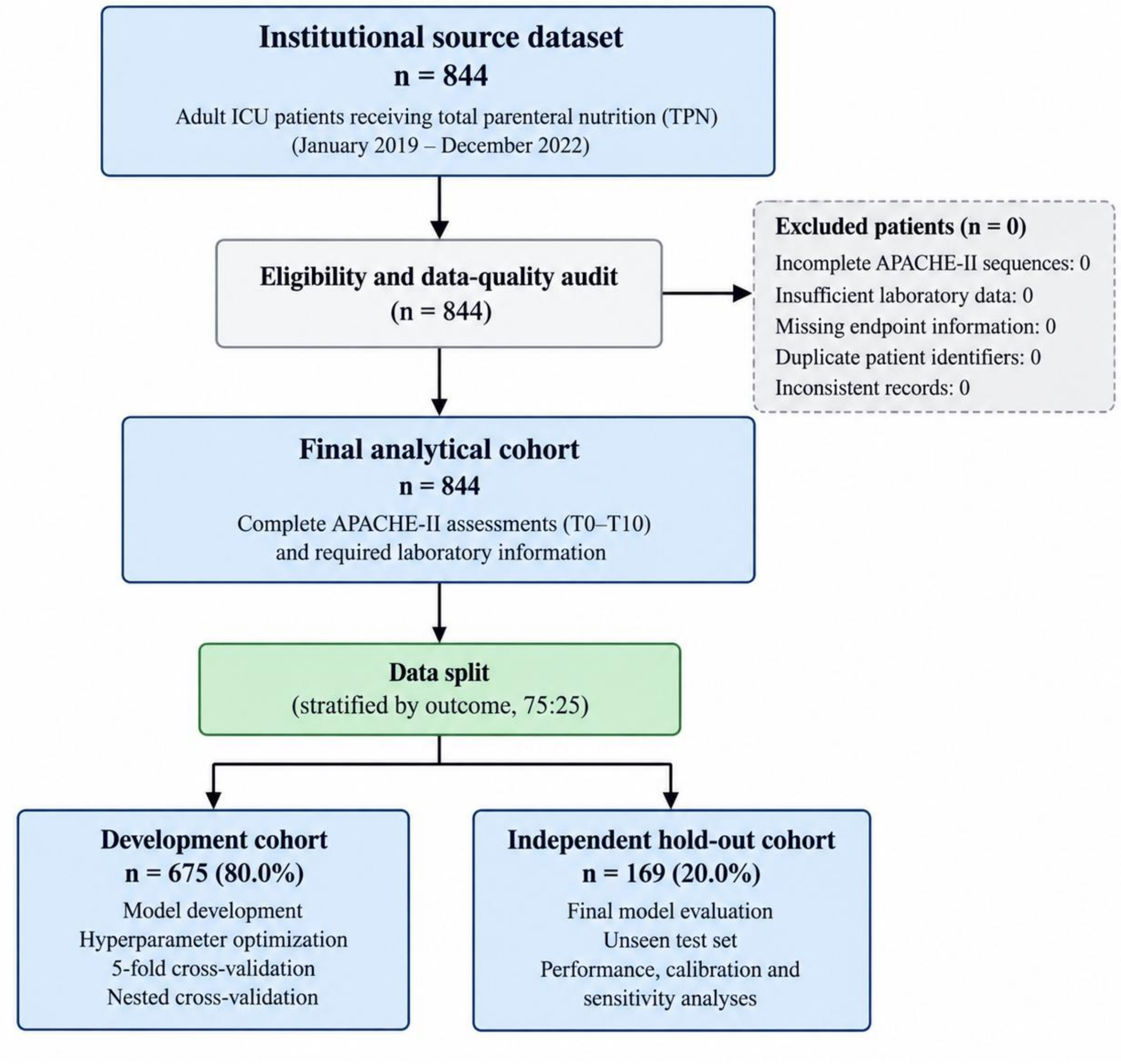

Supplementary Figure S1. Institutional cohort flow and data partitioning. The institutional dataset comprised 844 adult ICU patients receiving total parenteral nutrition (TPN). All patients had complete APACHE-II assessments from T0 through T10, including the endpoint-defining T10 assessment, and no patients were excluded during the final data-quality audit. The cohort was subsequently divided by stratified random sampling into a development cohort of 675 patients (80.0%) and an independent hold-out cohort of 169 patients (20.0%). The development cohort was used for model development, hyperparameter optimization, cross-validation, and nested cross-validation, whereas the hold-out cohort was reserved for independent model evaluation.

Note: Laboratory variables exhibited only low observation-level missingness (maximum 1.54%); therefore, "no exclusions for insufficient laboratory data" indicates that no patient was removed because of laboratory missingness. Missing laboratory observations were handled using training-derived median imputation within the modeling pipeline.
